# *Aronia melanocarpa* Flavonol Extract—Antiradical and Immunomodulating Activities Analysis

**DOI:** 10.3390/plants12162976

**Published:** 2023-08-17

**Authors:** Kseniya Bushmeleva, Alexandra Vyshtakalyuk, Dmitriy Terenzhev, Timur Belov, Evgeniy Nikitin, Vladimir Zobov

**Affiliations:** A.E. Arbuzov Institute of Organic and Physical Chemistry, Kazan Scientific Center, Russian Academy of Sciences, Arbuzov Str. 8, Kazan 420088, Russia; alex.vysh@mail.ru (A.V.); dmitriy.terenzhev@mail.ru (D.T.); belofftimur@mail.ru (T.B.); berkutru@mail.ru (E.N.); vz30608@mail.ru (V.Z.)

**Keywords:** fruits of *Aronia melanocarpa*, extraction, flavonols, antioxidant capacity, immunomodulatory activity, phagocytosis, oxidative stress damage

## Abstract

The study of *Aronia melanocarpa’s* (*A. melanocarpa*) biological activity is focused on obtaining the crude extract and separation of the flavonol compounds. The extraction and fractionation of *A. melanocarpa* fruits, followed by quantitative analysis, were accomplished using high-performance liquid chromatography and Darco G-60 filtering. This approach enabled the quantification of flavonoids within each fraction. The antioxidative, immunomodulating activities and cytotoxicity with respect to the lymphoblast cell line RPMI-1788 were studied. The flavonol extract of *A. melanocarpa* has been shown to have a high capacity to neutralize free DPPH and AAPH radicals in vitro. It also caused an accelerated ‘respiratory burst’ formation of neutrophils and an increase in the metabolic reserves of cells in rats exposed to cyclophosphamide. The reference solution (an equivalent quercetin-rutin blend) contributed to a decrease in lipid peroxidation, intensifying phagocytosis processes. The studied compounds demonstrated their low influence on the leukocyte blood profile in animals.

## 1. Introduction

Black chokeberry, *Aronia melanocarpa* (Michx.) Elliott is a shrub of the *Rosaceae* family native to North America that was transferred to Europe approximately a century ago. The edible parts of black chokeberry are mainly fruits that are widely used in the food industry for juices, syrups, jams, fruit teas and dietary supplements [1,2]. 

Aronia was used in Siberia and by American Indians as an elixir of youth that sharpened the mind and helped heal bone injuries. Frequently, Aronia was given to young women, especially during pregnancy, because it promoted strength development [3]. In the past, Aronia leaves were used in traditional medicine as anti-inflammatory, antiviral, antibacterial and antiproliferative agents [4]. Aronia leaf extracts have been used as a source of active ingredients for biopharmaceutical engineering [5]. Oral administration experiments revealed that the extract reduced the intensity of peroxidation of lipids and proteins induced by ascorbate and H_2_O_2_ in brain homogenates [6].

The biological activity of *A. melanocarpa* is known to correlate with the compounds of the polyphenol family, including anthocyanins, flavonols, flavanols, proanthocyanidins and phenolic acids. The content of phenolic constituents in *A. melanocarpa* is higher than that of most other berries and fruits investigated to date, which is consistent with its antioxidant potency in laboratory tests [7,8]. The antioxidant effect of black chokeberry extracts has been evaluated using different in vitro assays, such as inhibition of methyl linoleate oxidation [9], oxygen radical absorbance capacity [10], trolox-equivalent antioxidant capacity [11] and DPPH radical-scavenging activity [12].

Flavonols are one of the most representative classes of flavonoids, a group of natural derivates of 2-phenyl benzo-γ-piran, spread in the plant kingdom and playing numerous key roles in plant metabolism, ranging from ultraviolet and microbe attacks to plant and microbe interactions [13]. Flavonols are known to have various biological activities, including antioxidative [14], antihypertensive [15,16], immunomodulatory [17], antibacterial [18,19], anticarcinogenic [20] and antiviral [21]. Quercetin and rutin are two flavonols that are widely spread among plants and are often found in the daily diet, mainly in fruits and vegetables [22].

According to the literature, quercetin, genistein and flavones were not only able to suppress the production of reactive oxygen species (ROS), but also drove human neutrophil apoptosis [23]. Furthermore, flavonoids, especially flavone, flavonol, quercetin and rutin, significantly inhibited the migration of polymorphonuclear neutrophils [24]. These results revealed that flavonoids play an immunomodulatory role in protecting against the harm caused by neutrophils associated with continuous or uncontrolled inflammatory processes.

High immunomodulatory impacts of flavonols were associated with high antioxidative properties [25]. The adverse effects of synthetic drugs and the search for natural alternatives for therapy has led to an increased demand for complex flavonol actions to increase immunity [26,27].

The presence of immune modulators in higher plants has been extensively studied [28], but only a limited amount of immunomodulators of plant origin are known. These products may be developed into alternative aids in the treatment of disorders caused by an exaggerated or unwanted immune response, such as autoimmune diseases, allergies, glomerulonephritis, chronic hepatitis, etc. [29].

Even though *Aronia melanocarpa* extract is known to be a rich source of flavonoids capable of positively affecting the immune profile of animals, the derived *A. melanocarpa* flavonol extract still requires further investigation to comprehend the mechanism behind its curative properties. Previously, studies were carried out in isolation of the enriched flavonol fraction of *A. melanocarpa* using specialized sorbents; however, they did not differ in high sorption capacity, desorption coefficient or selectivity to flavonols.

Therefore, the main purpose of this research was to investigate the effect of the enriched flavonol fraction extract of *A. melanocarpa* using the Darco G-60 sorbent after its preliminary modification on the antioxidant system of the animal and on the phagocytosis of macrophages in vivo against the immunosuppression induced in rats by cyclophosphamide (CP).

## 2. Results

### 2.1. Chemical Composition and Antioxidant Activity of Extracts 

The total contents of sugars and natural antioxidants of the tested extracts are given in Table 1. The content of flavonoids in the original 70% ethanol extract of *A. melanocarpa* was lower by 2.3 times than the total anthocyanins, which is consistent with other studies [30].

According to other studies, the 70% ethanol used in the study was the best extractant due to its lower toxicity and increased extraction of polyphenols with lower selectivity [31,32,33]. The Darco G–60 treatment (a modified carbon chromatography sorbent) resulted in lower sugar content (down by 99.74%) compared to 70% ethanol chokeberry extract, with the total number of flavonoids increasing from 39.3 mg/g to 889.63 mg/g (on a dry basis). The quantity of anthocyanins experienced a further decrease, with the average content decreasing by 99.75%.

The flavonols are believed to be responsible for the antioxidant properties of the flavonol extract. The residual quantity of the anthocyanins in the extract will not provide a significant contribution to the antioxidative activity. The primary flavonols of two extracts of *A. melanocarpa* were identified by HPLC, based on their retention time in the column, spectroscopic characteristics and fragmentation pattern. Five individual flavanols have been identified (Table 2), with quercetin being the main component, accounting for 41% of the total number of flavonoids in the flavonol fraction and 43.8% for the ethanol extract. Kempferol, rhamnetin, isorhamnetin and dihydroquercetin were the remaining identified flavonols.

The study has also identified six glycosylated flavonols, which constitute approximately one-half of the total obtained flavonols. The major component was rutin (quercetin-3-O-rutinoside), which represented 34.6% in the ethanol extract and 35.4% in the flavonol extract of the total flavonoid content. The remaining compounds were hesperidin (hesperetin-7-O-rutinoside), quercetin-3-O-ramnoside, quercetin-3-O-galactoside, quercetin-3-O-glucoside and quercetin-3-O-hexoside (Table 3). 

Furthermore, the presence of phenolic acids, specifically chlorogenic and neochlorogenic, was detected in quantities of 0.517 and 0.439 mg/g on a dry basis, respectively. The acids of this kind were not detected in the ethanol extract, as their quantities were below detection limits, and their concentration took place in the flavonol extract.

The reversed-phase column of the hydrophilic HPLC enabled us to identify the remaining anthocyanins, which were present in low concentrations in the flavonol fraction. The identified compounds included cyanidin-3-galactoside, cyanidin-3-arabinoside and cyanidin-3-xyloside (Table 4).

After HPLC, the main compounds in the fractions were identified. Following the results of thin-layer chromatography (TLC), they had one predominant component (Fractions 4, 6–8, 12, 14, 20–22 and 24). Fraction 10 contained two compounds, whereas Fractions 16–17 contained three compounds. The inability to separate them is explained by the similar absorption properties of the material in use. The quantitative content of the identified flavonols is provided in Table 5. The fractional chromatograms are presented in Figure S1.

Since quercetin and rutin were major compounds in the flavonol fraction, constituting together 68.07% of the total extract mass, we believe that the antioxidant mechanism of the flavonol extract is based on these two compounds. So, a parallel study was performed, namely the evaluation of the antioxidant properties of the flavonol extract by separating this extract into distinct components primarily composed of quercetin (Table 5, Fractions 6–8) and rutin (Table 5, Fractions 20–22), both of high purity.

Electrospray ionization (ESI) mass spectra were obtained in the positive-ion mode (Figure S2), with the formation of the protonated ion [M+H]^+^, where H = 1 (mass of the proton) [34]. Each of the ESI mass spectra of Fractions 4, 6–8, 10, and 12 contained one primary ion peak. In addition to the primary signal associated with quercetin-3-O-ramnoside, there appears to be a mass fragmentation peak related to the quercetin fragment. Fractions 20–22 and 24 exhibit an additional ion peak of *m*/*z* 465, which is related to the quercetin-monosaccharide combination. Regarding Fractions 16–17, these yield a peak intensity as a harmonic of *m*/*z* 464.5. To ensure validation, the composition was analyzed using HPLC, since rutin and hesperidin (*m*/*z* = 610), as well as rhamnetin and isorhamnetin (*m*/*z* = 316), quercetin-3-O-galactoside and quercetin-3-O-glucoside (*m*/*z* = 464), together with quercetin-3-O-glucoside (M = *m*/*z*), all possess a similar molecular weight. The chromatograms of all the samples tested, together with the identification of their peak, were included as Supplementary Material (Figure S2). Treatment and extraction performed separately are helpful in revealing the mechanism of antioxidant properties and correlating them with the chemical composition and individual components of the flavonol extracts.

### 2.2. Extracts’ Effect on DPPH-Scavenging Activity 

The antioxidant properties of the *A. melanocarpa* berry flavonol extract were assessed using the well-known and convenient method of DPPH scavenging. It was also used to test certain flavonols for their capacity to scavenge DPPH radicals [35]. The radical scavenging activity was expressed as an EC_50_ value (mg/mL), i.e., as the concentration necessary to decrease DPPH concentration by 50%. The flavonol fraction, as shown in Table 6, exhibits superior AOA compared to the native *A. melanocarpa* berry extract and also quercetin and rutin solutions with equivalent concentrations, i.e., 9.3 and 10.4 times, respectively. For the ability to scavenge DPPH, the flavonol fraction was closest to quercetin, the antioxidant of natural origin. The half-maximal inhibiting concentration was 4.95 times higher. 

### 2.3. Chemiluminescent Activity of Investigated Compounds

The antioxidant activity of the investigated compounds was verified by the study of luminol-enhanced chemiluminescent activity. Chemiluminescence is a more attractive method for testing antioxidant activity due to its high sensitivity [36]. By adding the compounds to the chemiluminescent solution, one can intensify or diminish the light. It is assumed that the diminished light is a measure of antioxidant activity.

**Figure 1:**
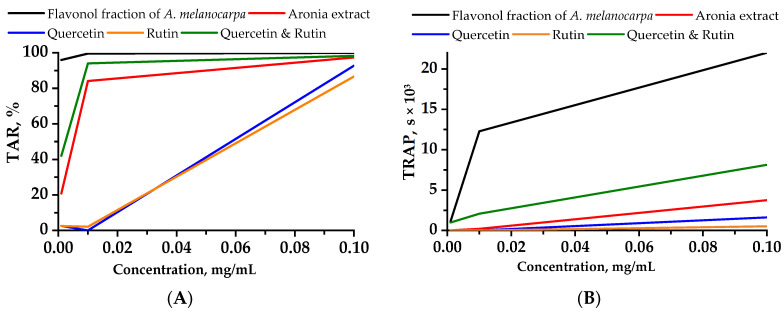
Chemiluminescent intensity attenuation (**A**) (TAR—total antioxidant reactivity) and time (**B**) (TRAP—total reactive antioxidant potential) vs. the concentration of the studied compounds. Values obtained from the quenching of luminol-enhanced chemiluminescence.

Among the extracts that have been examined, the flavonol fraction, whose active compounds are quercetin and rutin, has demonstrated significant inhibition of chemiluminescence, surpassing the fraction equivalent solution containing quercetin and rutin (Figure 1).

The prepared quercetin and rutin formulations did not follow the chemiluminescent inhibition time profile of the Aronia flavonol fraction. The inhibition time profiles for the concentrations of quercetin and rutin flavonols at 0.1 mg/mL were 20,375 and 21,490 s shorter than those of the flavonol fraction, respectively, and also 2131 and 3246 s shorter than the profile for the Aronia ethanol extract. The chemiluminescent activity profile determined by light emission suppression for the quercetin and rutin Aronia flavonol extracts and for the Aronia ethanol extract was slightly lower than that of the 0.01 mg/mL Aronia flavonol extract, namely by 5.5% and 15.5%, respectively.

### 2.4. Extracts’ Effect on Lymphoblast Normal Cells 

**Figure 2:**
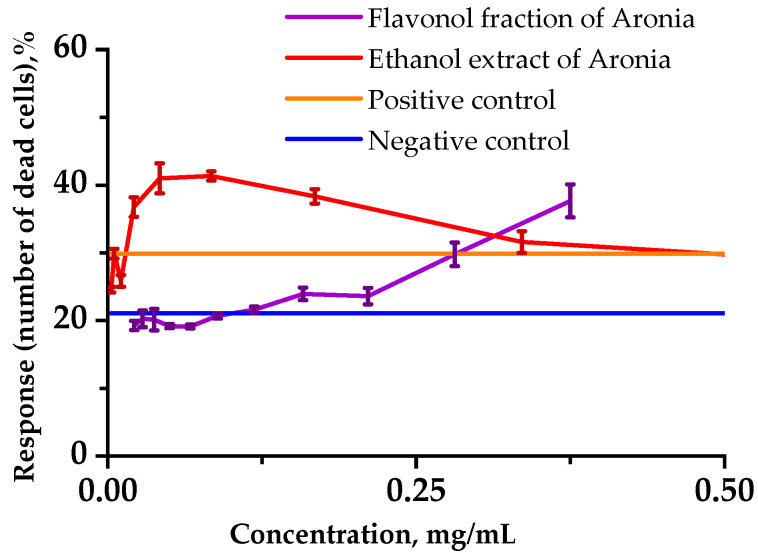
Influences of the analyzed substances on the viability of RPMI-1788 cells. Cells (1.0 × 10^5^) were incubated in 200 µL of culture medium without cyclophosphamide (CP) (negative control) and with 1.25 mg/mL CP (positive control) for up to 48 h. Viable cells were detected using Hoechst/PI fluorescence dyes and counted with a flow cytometer. Data represent the averages of three separate experiments. Error bars indicate the standard deviation.

In order to assess the toxicity of the extract and black chokeberry flavonol extract, we investigated the cytotoxicity using a human lymphoblastic RPMI-1788 cell line (Figure 2).

Analysis of the number of viable cells in each sample showed that incubating cells with Aronia ethanol extract at relatively low concentrations in combination with cyclophosphamide (1.25 mg/mL) caused a significant cytotoxic effect on normal human cells for 24 h in the culture medium. At 0.25 mg/mL and lower concentrations, the Aronia flavonol fraction had a protective effect on the viability of the lymphoblast cells under the stress induced by CP.

### 2.5. Hematology Study of Rats’ Peripheral Blood

The results of hematology studies provide important information about overall health, bone marrow activity, and possible intravascular effects, such as hemolysis.

**Figure 3:**
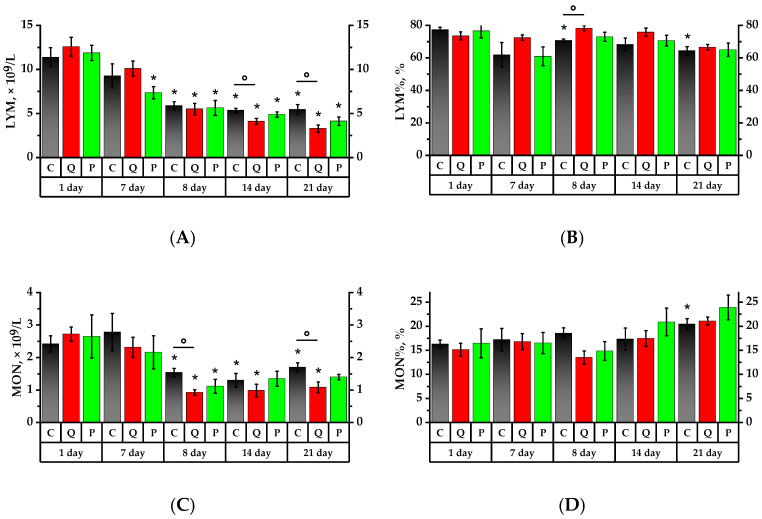

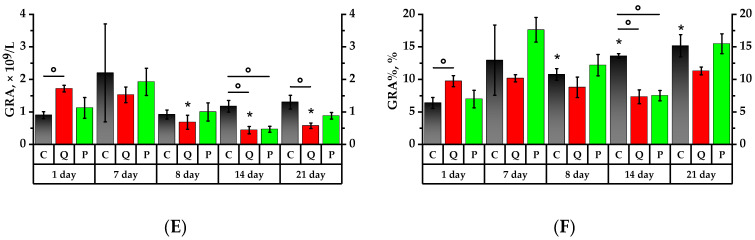
Leukocyte profile. (**A**) Lymphocyte (LYM) count; (**B**) lymphocytes to total leukocytes ratio (LYM%); (**C**) monocyte (MON) count; (**D**) monocytes to total leukocytes ratio (MON%); (**E**) neutrophil granulocyte (GRA) count; (**F**) granulocytes to total leukocytes ratio (GRA%). * *p* < 0.05, significance of differences compared to the first day of the experiment, ° *p* < 0.05, significance of differences between the animal groups.

**Figure 4:**
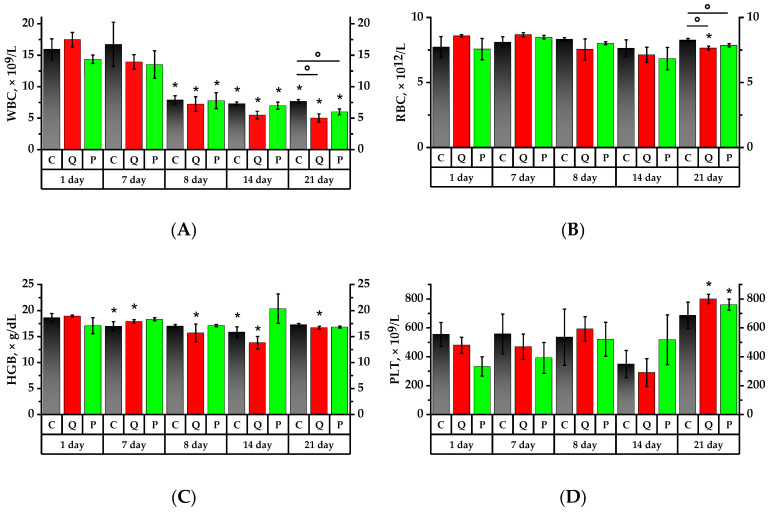

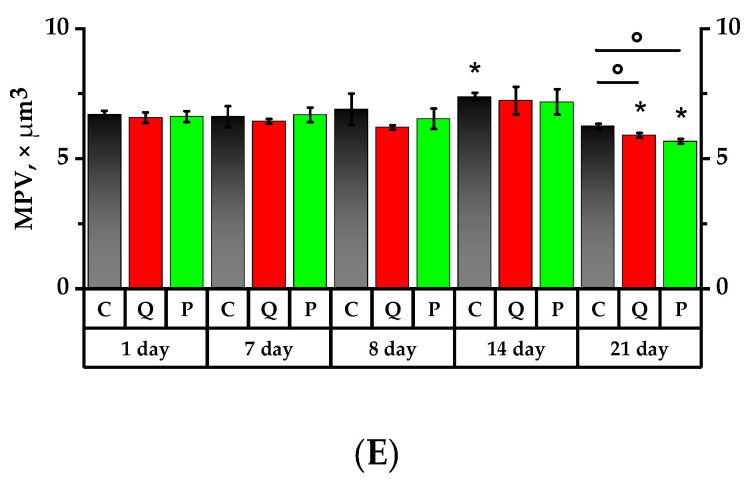
Main hematology results for whole blood from lab animals. (**A**) White blood cell (WBC) count; (**B**) red blood cell (RBC) count; (**C**) hemoglobin (HGB) concentration; (**D**) platelet (PLT) count; (**E**) mean platelet volume (MPV). * *p* < 0.05, significance of differences compared to the first day of the experiment, ° *p* < 0.05, significance of differences between the animal groups.

According to Figure 3 and Figure 4, the formulations under study did not show any significant impact on the leukocytes in rats administered proactively for a period of seven days. However, on the 8th day, after a 24-h exposure to CP, there was a significant leukocyte count decrease in all studied groups, namely: 2 times in the Control Group (Group C), 2.4 times in Group Q (quercetin at 50 mg/kg), and 1.85 times in Group P (a 50 mg/kg Aronia flavonol extract on a dry basis) compared to the values of the 1st day (*p* < 0.05). There were no significant changes in the subsequent seven days, but on the 21st day, the leukocyte number decreased in Groups Q and P. 

After consuming the Aronia flavonol fraction for seven days, there was a 1.6 lymphocyte decrease in Group P (*p* < 0.05). After a 24-h CP exposure, the lymphocyte absolute number decreased significantly in all investigated groups, namely: 1.9 times in the Control Group, 2.3 times in Group Q, and 2.1 times in Group P compared to the initial values of the 1st day. The lymphocyte ratio (LYM%) decreased by 1.1 times in Group C compared to the 1st day of the experiment, whereas Group Q exhibited a 1.1 higher LYM% compared to Group C (*p* < 0.05). Over the subsequent 7 days, there was a decrease in the absolute number of lymphocytes in Groups Q and P by 1.3 and 1.1 times, respectively, compared to the 8th day of the experiment, and by 1.3 times in Group Q in comparison with the Control Group (*p* < 0.05).

Monocyte count (MON) following CP administration decreased by 1.6 times in the Control Group, by 2.9 times in Group Q, and by 2.4 times in Group P compared to the values obtained on the 1st day (*p* < 0.05). On the 8th, 14th, and 21st days, the MON value in Group Q was 1.7 times lower than that of the Control Group (*p* < 0.05). On day 21, the monocyte ratio (MON%) increased significantly: 1.3 times in the Control Group, 1.4 times in Group Q, and 1.5 times in Group P compared to the values of day 1, while Group Q observed a decrease in MON that was 1.6 times lower than that for Group C (*p* < 0.05).

On the 1st day of the study, the granulocyte (GRA) count in Group Q was 1.5 times higher than that of the Control Group. The consumption of a mixture of quercetin and rutin over a period of 7 days increased the absolute number of granulocytes in rats by 1.7 times and also resulted in a granulocyte ratio (GRA%) increase that was respectively 1.1 and 2.5 times higher than the references in Groups Q and P. After a 24-h exposure to the CP, the absolute granulocyte count in Group P decreased by 1.9 on the 7th day, while GRA% increased by 1.7 times in both groups, i.e., the Control Group and Group P (*p* < 0.05). On day 14, Groups Q and P observed a decline in both GRA and GRA% against the Control Group (*p* < 0.05). On the 21st day, there was a 2.4-fold GRA% increase in the Control Group relative to the 1st day of the study. In Groups Q and P, it was, respectively, 1.2 and 2.2 times higher (*p* < 0.05), and the value of Group Q was lower than that of the Control Group (*p* < 0.05).

After a 24-h exposure to the CP, there was an erythrocyte (RBC) decrease in Group P by 1.1 times (*p* < 0.05). On the 14th day of the study, the RBC in Groups Q and P decreased by 1.2 times in both cases compared to the 7th day. In Group P, the RBC was lower than that of the 7th and 8th days (*p* < 0.05). On day 21, the RBC in Groups Q and P was, respectively, 1.09 and 1.05 times lower than that of the Control Group (*p* < 0.05).

On the 7th day of the study, hemoglobin (HGB) concentration in the Control Group and Group Q was found to decrease, respectively, by 1.09 and 1.06 times compared to the 1st day (*p* < 0.05). After a 24-h exposure to the CP, there was a 1.2-fold concentration decrease of hemoglobin in Group Q relative to day 1, and in Group P it was 1.1 times lower than that on the 7th day (*p* < 0.05). On the 14th day, the Control Group and Group Q observed, respectively, 1.2 and 1.4 times lower concentrations of hemoglobin than they were on the 1st day of the study, while the HGB in Group P tended to increase.

The mean platelet volume (MPV) of rats administered quercetin and rutin after one-day exposure to the CP decreased in relation to the 7th day and was 1.1 lower than that of the control group (*p* < 0.05). On the 14th day, MPV in Group Q increased by 1.1 times over the 8th day, whereas the MPV Control Group was 1.1 times higher than on the 1st day of the study (*p* < 0.05).

During the study, the PLT did not exhibit significant changes; however, it tended to increase in Groups Q and P exposed to the CP and tended to decrease in the Control and Group Q on the 7th day after its exposure.

### 2.6. Leukocyte Phagocytic Activity

**Figure 5:**
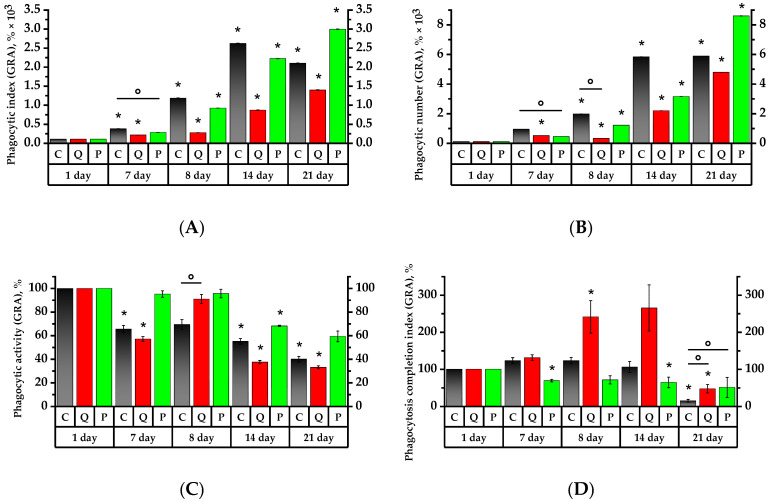
Values of phagocytic activity of rat peripheral blood neutrophil granulocytes. The corresponding baselines for each group on the first day of the experiment are taken as 100%. Values (phagocytic index (**A**), phagocytic number (**B**), phagocytic activity (**C**) and phagocytosis completion index (**D**)) are expressed in %. * *p* < 0.05, significance of differences compared to the first day of the experiment, ° *p* < 0.05, significance of differences between the animal groups.

**Figure 6:**
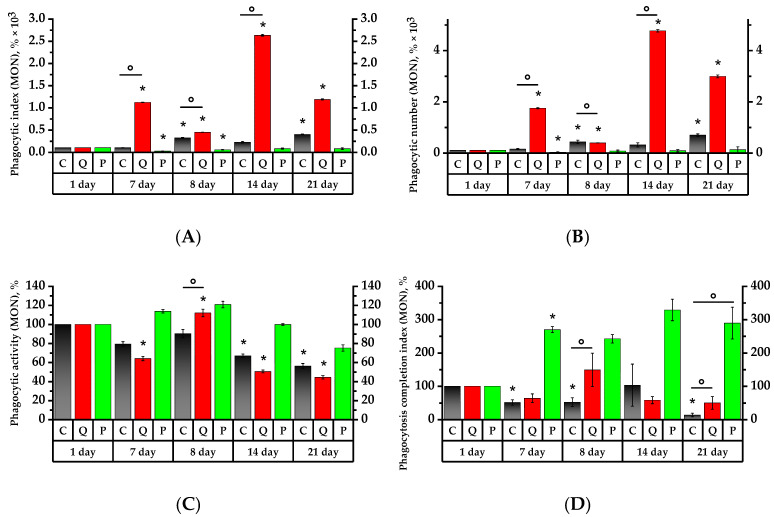
Values of phagocytic activity of rat peripheral blood monocytes. The corresponding baselines for each group on the first day of the experiment are taken as 100%. Values (phagocytic index (**A**), phagocytic number (**B**), phagocytic activity (**C**), phagocytosis completion index (**D**)) are expressed in %. * *p* < 0.05, significance of differences compared to the first day of the experiment, ° *p* < 0.05, significance of differences between the animal groups.

The results of the neutrophil and monocyte phagocytic activity study are depicted in Figure 5 and Figure 6. For convenience, the initial day’s data were taken at 100%, and the remaining percentage were calculated in relation to the initial day. 

On the 7th day, the granulocytes of the control group and Group Q demonstrated, respectively, 3.6 and 2.1 times higher phagocytic index (PI) and 9 and 5 times higher phagocytic number (PN) than on the 1st day of the study (*p* < 0.05). At the same time, the PI and PN of the granulocytes of Group P were, respectively, 1.9 and 2.25 times lower on the 7th day than the Control Group’s values of the same day (Figure 5). As for the Group Q monocytes, their PI and PN values increased, respectively, by a factor of 15 and 22, relative to the Control Group and the values on the 1st day; in Group P, the values decreased relative to the initial day of the study (*p* < 0.05) (Figure 6).

The 7th day revealed a 1.5-fold decrease in the phagocytic activity (PA) of granulocytes in the Control Group, while the PA of granulocytes and monocytes was 1.75 and 1.6 times lower in Group Q compared to the 1st day of the study, respectively. As for Group P, its PA of granulocytes and monocytes was, respectively, 1.4 and 1.3-fold lower than that of the control group on the 1st day of study (*p* < 0.05).

The phagocytosis completion index (PCI) revealed a 1.45-time decrease in the granulocytes and a 2.7-time increase in the monocytes in Group P compared to the 1st day of the study (*p* < 0.05). As for the PCI of monocytes in the Control Group, that became 15 times lower on the 7th day (*p* < 0.05).

The PI of granulocytes increased by 3.2 times in the control group and 3.3 times in Group P subsequent to a 24-h exposure to the CP, in contrast to the results prior to the aforementioned exposure. One day after CP exposure, both groups, C and P, revealed, respectively, 3.2- and 3.3-time higher PI values of the granulocytes compared to the ones before CP administration. The MONO PI increased by 3.2 times in the control croup and 2.4 times in Group P (*p* < 0.05). The GRA PI increased by 1.3 times in Group Q in comparison to the 7th day. The MONO PI, on the other hand, decreased by 2.5 times and was 3.4 times lower than the control group’s of the same day (*p* < 0.05). Regarding the results of the 7th day, the PN of granulocytes and monocytes, similar to PI, increased in the control group by 2.2 and 2.8 times, and in Group P by 2.75 and 2.6 times, respectively (*p* < 0.05). In Group Q, the PN of granulocytes and monocytes decreased by 1.6 and 4.4 times compared to day 7, respectively (*p* < 0.05).

As for the number of activated granulocytes and monocytes in Groups C and P, their PA remained below that of the control group on the 1st day. Compared to day 7, the PA of Group Q increased by 1.6 and 1.7 times, respectively, and was reliably higher than the PA of granulocytes and monocytes of the control group of the same day by 1.5 times (*p* < 0.05).

On the 8th day of the study, the Control Group did not reveal any significant changes in PCI, whereas Group Q increased the PCI of GRA and MONO by 1.8 and 2.3 times, respectively, compared to the values of the 7th day. These values were significantly higher than the PCI references of the same day, by 1.8 and 2.5 times, respectively.

Seven days after the introduction of CP, there was a PI increase in Group Q by 3.1 times and in Group P by 2.4 times compared to the 8th day (*p* < 0.05). Similarly, the PN of granulocytes became 7.2 times higher in Group Q and 2.6 times higher in Group P with respect to the 8th day of the study (*p* < 0.05). The PI and PN of monocytes in Group Q showed 5.9 and 12.1 times increases, respectively, while the PI of Control Group monocytes became 1.4 times lower compared to day 8 (*p* < 0.05). It is particularly remarkable that the PI and PN of monocytes in Group Q were significantly higher than the control values by 2.5 and 2.2 times, respectively. The PI and PN of monocytes in Group P became higher on day 8 in comparison to day 7, with no significant increase in these values afterwards.

The PA on day 7 after the introduction of the CP decreased significantly in Group Q by 31.1% for monocytes and by 28.8% for granulocytes with regard to the 8th day of study. On the 14th day, the PA of granulocytes in Group P was significantly lower by 10.3%, and the PA values of granulocytes and monocytes in the Control Group were respectively lower by 7.1% and 9% compared to the 1st day (*p* < 0.05).

Regarding PCI, there were not any significant changes in the Control Group on day 14, whereas the PCI of Group Q showed a 2.6-fold decrease with respect to day 8 of the study (*p* < 0.05), and the PCI of granulocytes remained as high as it was on day 8. Compared to day 8, the PCI of monocytes increased in Group P, whereas the PCI of granulocytes remained constant with the value on day 8 and declined by 1.6 times in comparison to day 1.

The PN of granulocytes in Groups Q and P increased by 2.2 and 2.7 times, respectively, on day 21, i.e., the 14th day after CP administration, compared to day 14 (*p* < 0.05). The PI values for granulocytes in Groups Q and P were significantly higher than those recorded on day 8 by a factor of 5.1 and 3.2, respectively (*p* < 0.05). The PN for monocytes in Group Q showed a 7.6-fold increase in comparison to day 8. As for Group P, this was 4.3 times higher compared to that of day 7 (*p* < 0.05).

The PA values remained the same on day 21, with the exception of Group Q, where the PA for granulocytes was found to be lower by 8.2% compared to the Control Group (*p* < 0.05).

The PCI for granulocytes and monocytes in Group Q was 2.9 and 5.6 times higher than in the Control Group, respectively. The same was true for Group P, which was higher by 3.3 and 4.2 times, respectively (*p* < 0.05). The PCI for granulocytes and monocytes in the Control Group decreased in comparison to day 14 by 7.2 and 7.8 times, respectively. The PCI for granulocytes in Group Q showed a 5.7-fold decrease compared to the 14th day (*p* < 0.05).

### 2.7. Study of Spontaneous and Activated Neutrophil Chemiluminescent Activity

Chemiluminescence, specifically luminol-enhanced, is widely used for phagocyte assessment [37]. Chemiluminescence (CL) is a quantitative measure of ROS generated by activated phagocytes [38]. However, the CL measures not only the phagocyte function of cells but also an intracellular oxidative metabolic response responsible for the generation of ROS. It is believed that CL is closely related to the bactericidal activity of the phagocytic cells [39]. This is the main reason for using the CL in clinical studies investigating the function of granulocytes.

**Figure 7:**
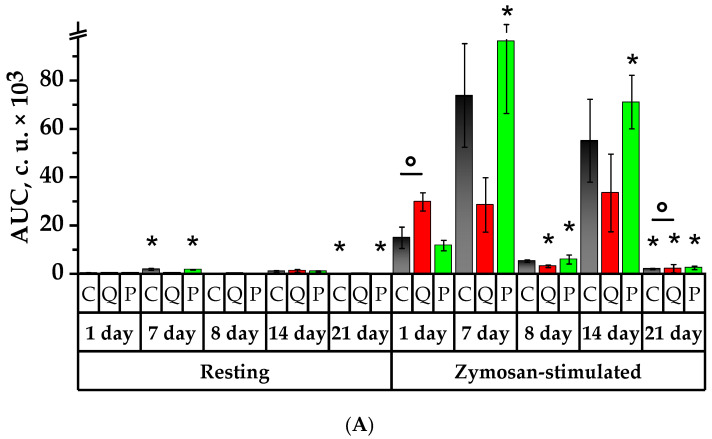

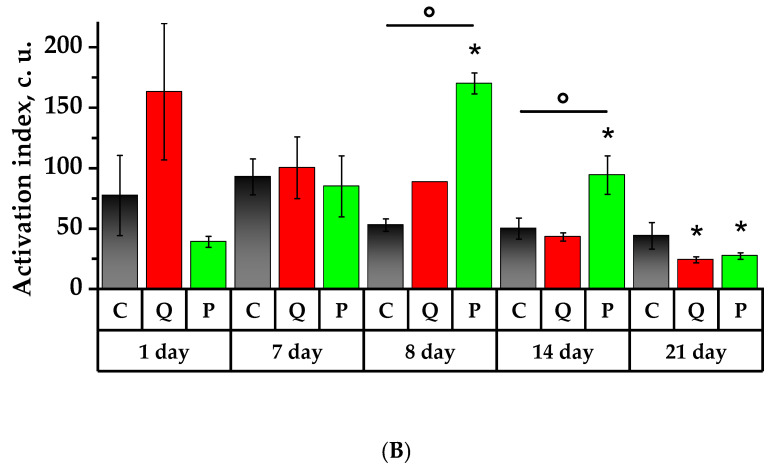
Values of spontaneous and zymosan-activated chemiluminescence of rat neutrophils: (**A**) area under the chemiluminescent curve (AUC, c.u.); (**B**) activation index (I_act_, c.u). * *p* < 0.05, significance of differences compared to the first day of the experiment, ° *p* < 0.05, significance of differences between the animal groups.

Over a span of 7 days, Groups C and P demonstrated a significant spontaneous intensity of the resting CL (I_max_) and of the area under the concentration-time curve (AUC), respectively, by 5 and 4.5 times for Group C and 5.6 and 5 times for Group P (Figure 7). In all studied groups, there was a slope growth of the CL curve relating to the data of the 1st day, namely by 4.3 times in Group C, 3.9 times in Group Q and 10.8 times in Group P. It was higher than the Control Group values by 2.2 times. The time showing the resting CL peak (T_max_) declined in Group Q and was lower than the control values by 2.8 times (Figure S3).

The neutrophils’ response to the CL reaction induced by zymosan was observed to be accompanied by a significant increase in I_max_ and AUC parameters in Group P, resulting in a 10.3 and 8.2 times increase, respectively, which was significantly higher than that of the Control Group. Compared to the 1st day, the kinetic rate of the zymosan-induced reaction grew spontaneously in Groups C and P. The time showing the induced CL peak of Group Q decreased by 1.2 times in comparison to the values recorded on the 1st day for the control group, and it was significantly lower in Groups Q and P with respect to the values of the 1st day in the Control Group.

On the 8th day of the study, one day after the CP administration, the spontaneous CL, AUC and slope were lower in Group C by 14.7 times, 18.6 times, and 6.4 times, while in Group P by 15.2, 22.3 times and 46.4 times, compared to the 7th day (*p* < 0.05). The value of T_max_ significantly declined in the control group by 2.7 times with respect to the values of the 1st day. The zymosan-induced CL demonstrated a significant decrease in I_max_, AUC and slope on day 8 in comparison to day 7 for all the groups in the study. It is noteworthy that the AUC of Group Q was significantly lower than that of the Control Group by 1.7 times, and there was a reduction in the time showing the CL peak, which was significantly lower than the control values by 1.2 times. The activation index (I_act_) experienced a significant increase in comparison to the initial day in Group P by 4.4 times, surpassing that of the control group by 3.2 times (*p* < 0.05). 

On day 14 of the study, the parameters of the spontaneous CL demonstrated higher microbicidal activity of the neutrophils in Groups C and P in comparison to day 8—I_max_ increased by 7.5 and 9 times, AUC—by 9.7 and 13.1 times, and slope—by 90 and 13.3 times, respectively (*p* < 0.05). The time showing the peak of the spontaneous CL, T_max_, decreased by 1.6 times in Group P in comparison to the 1st day, whereas it increased by 3.6 times in Group Q with respect to day 7 (*p* < 0.05).

On day 14, the results of the zymosan-induced CL demonstrated a significant increase in I_max_, AUC, and slope in comparison to the values of the 8th day, namely by 12, 10.5 times, and 13.8 times in Group C; by 8.8, 10.8, and 6.8 times in Group Q; and by 13.1, 6.4 and 13.9 times in Group P, respectively (*p* < 0.05). The peak time of the induced CL declined in all studied groups in comparison to day 1 and declined in Groups C and P in comparison to day 7. The opposite case was observed in Group Q, which showed a 1.1-fold increase in T_max_ compared to the 8th day. On day 14, there was a diminution in I_act_ values in Groups C and Q by 1.9 and 2.3 times, respectively, in comparison to the values recorded on the 7th day. Compared to the 1st day, Group P observed a higher I_act_ by 2.4 times, and it was significantly higher than the values obtained on days 8 and 14 for the Control Group by 1.8 and 1.9 times, respectively (*p* < 0.05).

On the 21st day of the study, the background CL revealed a significant diminution in the bactericidal activity of the neutrophils in Groups C and P compared to the 1st, 7th and 14th days in terms of parameters such as I_max_ and AUC. Similarly, the kinetic rate of the reaction exhibited a decline in Groups C and P in comparison to days 7 and 14, whereas in Group P it was higher than that of the 8th day. In Group P, the T_max_ of neutrophils was lower than that of the initial day by a factor of 2.4 (*p* < 0.05). The induced CL also revealed diminished values of I_max_, AUC, and slope in all the groups under the study compared to days 1, 7, and 14. However, the values of Group Q exceeded the references in the following manner: I_max_ was surpassed by 1.6 times, AUC by 1.1 times, and slope by 2 times (*p* < 0.05). On day 21, the T_max_ value was lower than the respective value of the initial day for the groups under the study. The value of T_max_ in the control group was 1.11 times lower compared to day 8, whereas in Groups Q and P, this value was 1.15 and 1.05 times higher compared to day 8 (*p* < 0.05). The T_max_ in Group P was 1.13 times longer than that in Group C (*p* < 0.05). On day 21, the I_act_ in Groups C and Q decreased in comparison to the 7th day by 2.1 times and 4.2 times, respectively. In Group P, the I_act_ values were 1.4 times, 6.2 times, and 3.5 times lower than the values obtained on days 1, 8 and 14 (*p* < 0.05).

### 2.8. Lipid Peroxidation Study

**Figure 8:**
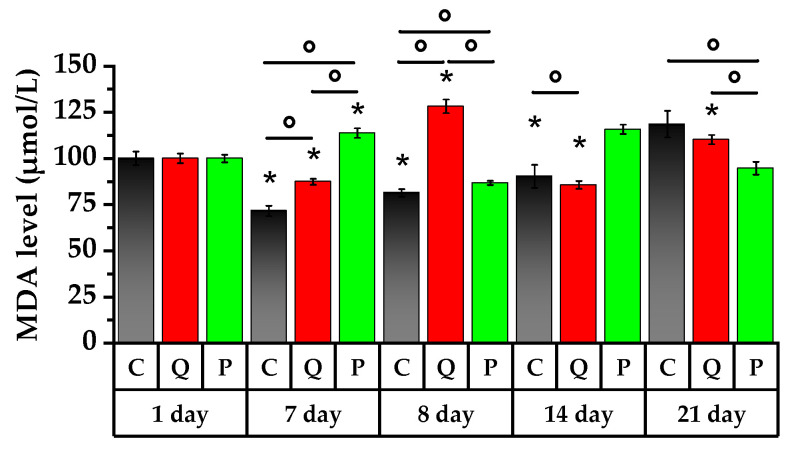
MDA level of peripheral blood of rats. * *p* < 0.05, significance of differences compared to the first day of the experiment, ° *p* < 0.05, significance of differences between the animal groups.

Rat lipid peroxidation (LP) was investigated to determine the substances’ impact on the antioxidant system of the animals (Figure 8).

On the 7th day of the study, blood samples of the rats from the control group and Group Q demonstrated a significant decrease in MDA levels of 28% and 13%, respectively. In contrast, MDA levels in rats taking Aronia flavonols increased by 14%.

A single dose of the CP on the 8th day of the study caused an accumulation of the LP product by 14% in the control group and by 47% in Group Q with respect to day 7 of the study. This is a demonstration of the adaptive responses of the body to toxic exposure. In contrast, the group of rats receiving Aronia flavonols showed a 23% decrease in MDA (*p* < 0.05).

Over the course of the subsequent 7 days, both the control group and the group of rats receiving the Aronia flavonol fraction experienced an increase in their MDA levels of 11% and 33%, respectively. The MDA level in Group Q experienced a decrease of 33% (*p* < 0.05).

On the 21st day, the rats receiving the Aronia fraction had lower LP products in their blood by 18% compared to the 14th day of the study while the LP level in Group Q increased by 29% against day 14 (*p* < 0.05). Furthermore, the LP intensification in the control group was only detected on the 21st day of the study, exhibiting a significant increase of 46% compared to the 8th day. In contrast, in Group Q, the highest level of LP products was observed on the 1st day after administration of the CP, and in Group P, on the 7th day after administration of the latter.

## 3. Discussion

In this study, the effect of the flavonol fraction of *A. melanocarpa* fruits’ extract on neutrophil activation, phagocytosis, and microbicidal response, as well as antioxidant potential, was investigated and compared with a mixture of major components such as quercetin and rutin in concentrations equivalent to the flavonol fraction.

In this work, modified Darco G-60 carbon was used to obtain an enriched flavonol fraction of *A. melanocarpa* berries. Thus, in the works for the isolation, separation and purification of flavonols, seven macroporous resins are mainly used: X-5, D4020, LS-305, LS-46D, D101 and NKA-9, which have different physical properties (Table 7). At the same time, the high sorption capacity of carbon sorbents with respect to phenolic compounds is widely known, the negative point of which, in the case of the isolation of natural compounds, is a low desorption coefficient and low selectivity with respect to specific groups of polyphenols. This factor in coal is eliminated by chemical activation and functionalization.

Among synthetic macroporous resins, the adsorption capacity and desorption coefficient of the non-polar X-5 resin were higher than those of other artificially produced sorbents. When used, the initial carbon Darco G-60 had a higher sorption capacity relative to the X-5 sorbent (2.1 times higher), but was distinguished by a low desorption coefficient (44.8% lower) and selectivity to flavonols (by 12%). After the modification procedure, the obtained carbon adsorbent has a 3-fold increase in the adsorption capacity for quercetin, the desorption coefficient of the relative macroporous resin X-5 increases by 2.4%, and the selectivity by 10%.

Results of luminol-enhanced CL testify that the Aronia flavonol fraction can inhibit luminol oxidation by AAPH suppression, absorbing alkyl or peroxyl radicals, or reducing oxidized AAPH.

The results of the cytoprotection analysis against the CP effect made it possible to suppose that the Aronia flavonol fraction has a beneficial impact on normal human lymphocyte cells at lower concentrations, whereas it exerts a toxic effect at higher concentrations. There appear to be optimum concentrations for flavonoids to act as prooxidants [40].

Previously, it was found that flavonoid glycosides had been found not to be incorporated into cells [41]. That is to say, rutin barely penetrates into cells due to its higher hydrophilicity, and therefore may have no biological effect. High concentrations of flavonoids, on the other hand, might be risk factors for endothelial injury. Our results on RPMI-1788 cell line suggest that cytotoxicity of flavonoids in high concentrations is due to their intracellular ability to generate ROS [40].

It is acknowledged that there are both agonistic and synergic interactions between distinct flavonoids within a mixture [42]. In the first case, the antiradical activity of the flavonoid mixture is less than the sum of antiradical activities of individual components, in the second case it is greater. Synergy and antagonism also depend on the method used to determine the antiradical activity of the substances under investigation. When studying the ability of flavanols to intercept free radicals, it is shown that the interaction of quercetin and rutin is antagonistic in this method [43]. However, in this work using the DPPH method, as well as luminol-enhanced CL, the interaction of quercetin and rutin in Aronia flavonol fraction revealed a high synergistic effect. There is also evidence that joint presence of quercetin, rutin and resveratrol in the extract enhanced the antioxidant effect of each substance [44]. Total flavonoids of the Aronia flavonoid fraction, as well as the activity of DPPH radical scavengery and chemiluminescent activity correlate, that is, the majority of Aronia flavonoids are involved in antioxidant activity.

Previously, the authors have found that rutin plays a facilitating role in CP intoxication [45]. The introduction of rutin appeared to have intensified the phagocytosis process, as evidenced by the dose-dependent enhancement in carbon particle clearance [46].

The analysis of hematologic parameters of animal blood revealed that the intake of the Aronia flavonol fraction for 7 days did not result in reliable changes in the total leukocytes of rats. At the same time, a decrease in the relative number of leukocytes and an increase in granulocytes were observed in all groups, mostly in Group P. At the same time, after the Control Group and the animal group were given a quercetin-rutin blend, hemoglobin concentrations decreased within the normal range.

Neboh and Ufelle, the authors [47] have shown that CP treatment induced normocytic normochromic anemia; it may be associated with the property of alkylating anticancer drugs that interfere with DNA synthesis and division of cancer cells as well as normal tissue proliferation, including bone marrow progenitor stem cells, resulting in pancytopenia. As for the leukocyte picture, CP caused a significant decline in total leukocyte, lymphocyte, and monocyte counts [48]. Such conclusion can be attributed to CP toxic metabolite phosphoramide mustard which was considered to be a myelosuppressive agent by reacting with bone marrow cell DNA, causing its dysfunction and resulting in loss of stem cells and bone marrow inability to regenerate new blood cells [49]. In addition, the CP inhibits the activity of splenic natural killer cells and suppresses mRNA expression of T-bet/GATA-3 (Th1/Th2 transcription factors) in splenocytes, resulting in lymphocyte-deplete in peripheral blood and tissue [50].

Immunodeficiency state was observed equally in all animal groups in 24 h after CP exposure, as the total number of leukocytes decreased, while the leukogram shift is maintained towards an increase of granulocytes and decrease of lymphocytes, although there was a decrease in the granulocyte percentage in all groups. After taking a mixture of quercetin and rutin, the leukogram of rats was noted to change towards an increase in lymphocytes as well as a decrease in hemoglobin and mean platelet volume. The rats taking the Aronia flavonol fraction had a change in the leukogram towards an increase of granulocytes as well as a decrease of the red blood cell within normal range. Neutrophilia in the control group and Group P may have been the result from activation of proinflammatory mediators (IL-1β, IL-6, and TNF-α) that induced the production of granulocyte-macrophage colony-stimulating factor which has an important role in neutrophil differentiation and maturation [51]. In addition, CP administration induced IL-5 upregulation by Th2-type cells in the lymph node and spleen, resulting in eosinophilia [52].

On the 14th day of the experiment, after 7 days of CP exposure, Group Q and Group P had a decrease in the granulocyte percentage compared to the Control Group and a decrease in the number of erythrocytes; Group Q had a decrease in hemoglobin concentration and platelet volume; however, the average platelet number increased. The authors, Murphy K.J. et al. showed that flavonol compounds, in particular catechin, are able to inhibit platelet function due to their antioxidant activity [53]. It is assumed that this is due to the ability of polyphenols to scavenge H_2_O_2_, produced during the cascade of arachidonic acid metabolism, resulting in platelet aggregation [54]. Platelet count increased in Group P, probably due to thrombocytopoiesis processes.

On the 21st day of the experiment, an increase in the granulocyte content was observed as well in the Control Group and Group P; there was an increase in the number of monocytes in all groups. Macrophages and monocytes are considered critical cells of the inflammatory phase [55]. These cells are involved in microbial phagocytosis, cellular debris and damaged matrix removal by secretion of matrix metalloproteinases. In addition to their role as the main source of cytokines and growth factors, monocytes stimulate fibroblast proliferation [56]. In the experimental Group Q and Group P, platelet level increased on the 21st day, but RBC and HGB levels decreased. MPV decreased in all the groups under investigation.

Analysis of phagocytosis activity revealed an increased number of an engulfed microorganisms in phagocytized blood monocytes in the group of animals receiving a quercetin–rutin blend on the 7th day of preventive treatment, as well as after immunosuppression therapy. The number of microorganisms engulfed in phagocytosing granulocytes was found to be increased in the control group on the 14th and 21st days of the experiment, as well as in the group receiving flavonol fraction, reliably on the 14th day of immunosuppression therapy, and exceeded the above number of microorganisms in phagocytosing granulocytes in the Control Group.

It is worth noting that the number of active phagocytized monocytes and granulocytes increased reliably in the group of rats receiving a quercetin–rutin blend in response to CP introduction, but it decreased during the period of further therapy. The number of phagocytized granulocytes in Group P decreased on the 14th and 21st day of the experiment relative to the initial values.

The study of phagocytosis completion index showed that while the digestive capacity of monocytes increased on the 7th day of receiving the Aronia flavonol fraction, the digestive capacity of granulocytes decreased in a compensatory way. At the same time, the PCI in the Control Group decreased compared with the initial values. In response to CP introduction, the PCI of granulocytes and monocytes increased in the group of animals receiving a blend of quercetin and rutin; after 7 days, the PCI of monocytes decreased. On the 21st day, the PCI of granulocytes and monocytes in the experimental Group Q and Group P was higher than that in the control group.

As can be seen from the above, the capacity of the Aronia flavonol fraction obtained in this work to activate the digestive capability of monocytes was mainly manifested in healthy animals. At the same time, when animals received a quercetin and rutin blend, the digestive capacity of neutrophils and monocytes was activated, both in animals in good health and under immunosuppression. Additionally, intake of quercetin and rutin reduced PCI in monocytes already on the 7th day and in granulocytes on the 14th day after CP introduction.

Rutin (quercetin glycoside) showed less antioxidant activity than its aglycone equivalent (quercetin). The amount of sugar moieties in flavonoid (in the resulting flavonoid glycosides) and their position, all play an important role in antioxidant activity. Aglycons are often found to have more antioxidant activity than their glycoside counterparts. Even though glycosides are generally considered to be weaker antioxidants than aglycons, a glucose moiety may sometimes improve bioavailability [43]. Quercetin is a strong inhibitor of neutrophil oxidative metabolism. However, in addition to scavenging free radicals, other mechanisms may mediate the action of these compounds on neutrophil effector functions. Flavonoid anti-inflammatory mechanisms of action include inhibition of respiratory burst and degranulation of neutrophils, arachidonic acid metabolism modulation, proinflammatory cytokine and chemokine gene expression modulation, interaction with cellular membranes and intracellular signaling proteins, as well as free radical scavenging [57].

According to the immunosuppressive experiment of rats, flavonoids such as myricetin, apigenin and hesperidin were found to enhance the macrophage phagocytosis levels, lymphocyte proliferation, and cytotoxicity of natural killer cells. They also reduced the toxicity of chemotherapeutic drugs and had a synergistic effects in tumor therapy [58]. The multi-target pharmacological properties of the flavonoids were able to facilitate their interaction with a variety of immune cells and immune mediators, thereby regulating the tumor immunosuppressive microenvironment at various levels, restarting immune surveillance, enhancing immune response to tumor cells, and even directly killing tumor cells. This approach has the advantage over other drugs targeting tumor-associated macrophages [59,60,61,62].

The study of luminol-dependent chemiluminescence of neutrophilic granulocytes offered the possibility to characterize NADPH-oxidase activity state in neutrophil granulocytes for the intact and immunosuppressed animals. Following the analysis parameters of spontaneous and zymosan-induced chemiluminescence (I_max_ and AUC), a reliable increase in bactericidal activity of phagocytizing cells was observed in association with animal intake of the flavonol fraction of *A. melanocarpa* in comparison with the control group. During the administration of quercetin–rutin blend to healthy animals, the rate of “respiratory burst” development increased, thereby indicating the elevated activity of NADPH-oxidase in neutrophil granulocytes in healthy animals.

The immunosuppression factor was found to reduce the neutrophils’ capability to activate ROS on the 8th day in all groups under the study. A day after CP introduction, a zymosan-stimulated “respiratory burst” in Group Q shortened the CL peak time, decreased the AUC, and the indicators were reliably below the control values. The activity can be explained either by the acceptance action of the mixture on ROS, or by functional changes undergoing by the cells in the presence of the mixture. The latter assumption is much more likely, since zymosan-stimulated cells are more inhibited than non-stimulated ones. Moreover, since the inhibition of the mixture on CL emission is much more obvious in the presence of zymosan, inhibition of oxygen release by phagocytes can depend on the mixture involvement in the zymosan dependent activation of protein kinase C [63]. On the 14th day of the study (7 days after CP introduction), both the neutrophil granulocytes in relative dormancy and the respiratory burst stimulated additionally with the zymosan increased the CL time in Group Q. On the 8th and 14th days of the experiment the activation index increased in the group of animals fed with Aronia flavonol fraction. The increased value of the activation index indicates that neutrophils have metabolic resource for functional activation. The absence of similar changes in antigenic zymosan-stimulated cells of Group Q suggests a limitation in the rate at which NADPH oxidase is activated, which is influenced by the cell’s metabolic resource. The reduction of spontaneous CL peak time in Group P on the 14th day demonstrates the capacity of the cell metabolic system to produce a high level of superoxide-radical.

LP process, as a consequence of ROS’ action, causes cell death due to disruption of cell membrane integrity [64]. In this work, the LP index was used for the estimation of the antioxidant activity of flavonols to find the compound responsible for antioxidant activity in vivo. The results of the analysis revealed a low level of LP and free radical formation in the group of control animals and Group P after CP introduction compared to Group Q. Furthermore, after seven days of feeding with the Aronia flavonol fraction, there was a tendency for MDA to decrease, which indicated the beneficial effect of flavonol antioxidants to prevent oxidative damage in immunosuppressive animals in vivo. A reduction in the MDA to the initial values was observed in Group Q on the 7th day after CP introduction and in Group P on the 14th day of flavonol fraction therapy after CP introduction.

In this study, the effect caused by Aronia berry extract rich in flavonols was found to be negligibly effective in reducing circulating levels of MDA both in healthy animals and immunosuppressive rats. In vivo, the flavanol fraction of Aronia appeared to show little antioxidant activity due to its limited bioavailability [65].

The authors [66] noted that quercetin and rutin showed better results in reducing the damage associated with LP and ROS processes under oxidative stress. In this study the intake of quercetin and rutin solution resulted in a short-term increase in the content of LP products of immunosuppressive rats and decrease of this content after 7 days of appropriate treatment.

The correlations between chemical structure and antioxidant capacity have showed following circumstances to be crucial for flavonoids to exhibit potent antioxidant activities: the hydroxyl group at the C-3 position of C ring, a double bond between carbons C2-C3, and the carbonyl group at C-4 position of C ring. Hydroxyl groups on C-5 and C-7 positions of the A ring as well as C-3′ and C-4′ of the B ring also enhance their antioxidant activities. Quercetin and rutin have all these structural features. Despite the fact that sugar moiety of rutin reduces the antioxidant activity of adjacent hydroxyl groups due to steric hindrances, it can be hydrolyzed by the intestinal flora to its corresponding aglycone quercetin and can also maintain its in vivo effects [67].

Thus, the capability of rutin and quercetin to react with superoxide ions and lipid peroxide radicals as well as maintain their antioxidant function indicates the possibility of using these flavonoids to fight against free-radical pathologies.

## 4. Materials and Methods

### 4.1. Sampling

**Figure 9:**
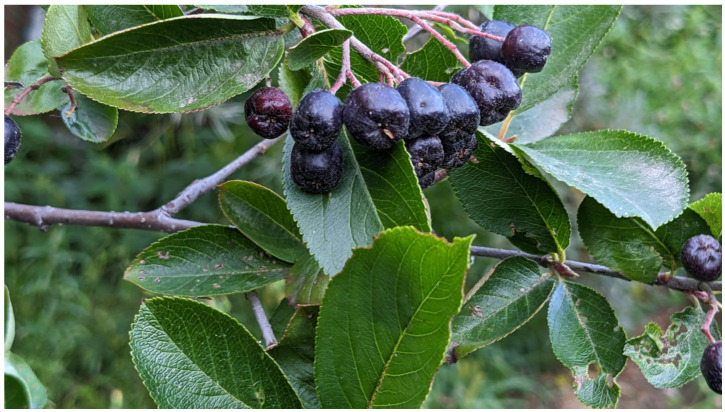
Fruits of black chokeberry variety “Black Pearl”, Ivanovo region.

As a raw material we used the berries of the Black Pearl chokeberry bush that were collected and characterized in our previous study [33]. The mature Aronia berries in umbel form were gathered in the Shujskie Yagodi farm (Ivanovo region) during the harvest season in September 2022 (Figure 9). The berries had max. diam. 0.9 cm and mass average 1.1–1.3 g. Berries were randomly collected from ten bushes, in the amount of 0.5 kg per plant. At the time of berries harvest the chokeberry bush was 6 years old. The berries were washed with distilled water to remove dust and contaminants and dried with cotton cloth and stored in the freezer at −35 °C for further research.

### 4.2. Preparation of a Modified Carbon Sorbent 

A Darco G-60 carbon (Thermo Fisher Scientific Inc, Waltham, MA, USA) dried to constant weight at 120 °C, was crushed to a particle size of approximately 0.5–1 mm in LM 202 mill (LLC Plaun, Moscow, Russia). The required particle size was obtained by carbon sieving. The carbon was modified in a laboratory muffle furnace in the form of a 5-L ceramic chamber with a closed heating element, which provides for reaction zone heating to the temperature required for initiation and activation (700 °C).

The temperature in the reaction zone was controlled by automatic control unit. Both the superheated steam and carbon dioxide, with a temperature of 210 °C, were fed into the activation area. The carbon activation was carried out during 1.5 h with subsequent cooling by dry argon flow. After modification, the absorbing capacities were found to increase (Table 8). The iodine index was determined by the amount of absorbed iodine using the titration method [68]. The number of end carboxyl groups prior to and after activation was determined by potentiometric and amperometric methods [69].

Due to its nature and affinity, the obtained carbon enables to absorb mainly polyphenol compounds.

### 4.3. Extract Preparation

This study examined the antioxidant properties in relation to the chemical composition of the substance. The first phase of work was to obtain ethanol extract from frozen berries. The frozen berries of aronia were ground into a paste in a laboratory mill with the forced cooling of the milling tube (KN 195 Knifetec, Labtec Foss, Hellirod, Denmark) and extracted by a three-stage maceration in flat-bottomed conical flasks pre-purged with argon. The extraction was carried out by a 70% ethanol solution with a biomass-solvent ratio equal to 1:5 and mixed at 500 rpm using an automatic magnetic mixer (Ohaus, Guardian 7000, Parsippany, NJ, USA) at 45 °C for 1.5 h with a constant flow of dry argon to avoid oxidation of active compounds [33]. The obtained homogenate was centrifuged to precipitate large particles and suspension at 11,000 rpm for 15 min at +5 °C to avoid oxidizing processes (H3-18KR centrifuge, Hunan Kecheng Instrument EquipmentCo, Ltd., Changsha, China). The extract was concentrated on a rotary evaporator (LabTex Re 100-Pro, Labtech Company, Moscow, Russia) over the water bath at 30–32 °C and 13.3 mbar until the solvent is completely removed. The dried extract was stored in a medical freezer at −35 °C for further analysis. Later, the extract was used as a reference sample. The extract was dissolved in 1.3-propylene glycol to produce 1% solution for the studies.

### 4.4. Preparation of Flavonol Fraction

The 100 g of frozen and crushed of *Aronia melanocarpa* berries were extracted using the maceration method with 90% ethanol solution and 1.3% potassium dihydrophosphate to create a buffer system with pH = 5.5 for 3 h at 45 °C in a water bath at a ratio of 1:8.5. The obtained extract was purified in the way similar to obtaining 70% ethanol extract and concentrated in vacuum (13.3 mbar) 12 times [70]. The method of column chromatography was used to purify and recover the flavonol fraction [34]. Darco G-60, a modified carbon chromatographic sorbent, with particle size distribution from 0.7 to 1.0 mm was used as the stationary phase.

The sorbent material was placed in a glass column (3 × 55 cm). The bottom of the glass column was covered with a layer of calcinated glass wool followed by a layer of a modified sorbent with a height of 30 cm and then a layer of glass wool over it for integrity of the absorbing layer. The column was washed by the elute selected by TLC method, consisting of the solvent system of ethanol: ethyl acetate (1:9). Then the investigated extract was added and seven eluted fractions of 50 mL each were collected. Using the same mobile phase in the TLC, it was determined that the distribution coefficient R_f_ (0.57) for fractions from 3 to 6 was similar. Thus, these fractions were combined. A 254-nm-wavelength UV light was used to visualize the spot [71]. The total volume of the fraction obtained was 200 mL, then it was dried until the elute evaporated. Dry flavonol fraction of *Aronia melanocarpa* had the form of a grey-yellow crystalline powder with pH = 7.15–7.25. For the studies, the dried flavonol fraction was dissolved in 1.3-propylene glycol to produce 1% of the solution.

### 4.5. Chemical Composition Analysis

The total flavonoid content was determined using Stankovich method [72]. The extract and flavonol fraction (0.5 mL each) were placed in different test tubes, and then 10% aluminum chloride (0.1 mL), 1 mole of potassium acetate (0.1 mL), 80% methanol (1.5 mL), and distilled water were added to each test tube. The resulting solutions were mixed. The reference solution was prepared in the same manner, except that a known concentration rutin solution was used instead of the extract. All test tubes were incubated at room temperature for 30 min. The optical density was determined at λ_max_ = 415 nm using a stationary wavelength scanning spectrophotometer (UV/VIS spectrometer T7DS). Flavonoid concentrations were expressed in milligrams of the rutin equivalent per gram of dry extract (Rut mg/g).

The residual anthocyanins were determined by spectrophotometric differential method in terms of the equivalent amount of cyanidin-3-O-glucoside (mg) per gram of dry extract [73]. The total sugar content was determined by spectrophotometric method at 520 nm using a 5% water solution of phenol and concentrated sulfuric acid. A solution of xylose was used as a standard [74]. The results of analysis are listed in Table 1.

### 4.6. Chromatographic Analysis of Flavonol Fraction Extract 

The combined flavonol fraction was subjected to column chromatography to separate individual flavonols and flavonols in glycosilated form available in *Aronia melanocarpa*. The glass wool thin layer and densely filled absorbent MSM-41 (ACS Material LLC., Pasadena, CA, USA) with particle size of 100–1000 nm and size pores of 3.4 nm was placed on the bottom of the glass chromatographic column (2 × 45 cm).

Using column chromatography, 25 elute fractions were collected with the help of two solvent systems, such as ethyl acetate- acetic- acid-water (90:3:7)—I and methanol—formic acid—water (75:1.5:23.5)—II. The solvent systems were selected by the method of TLC. The UV irradiator of TLC 254/365 (Petrolaser NPO, Saint Petersbeurg, Russia) with wavelength of 254 nm was used to visualize the spots. Using these mobile phases, the fractions of 12 mL were collected; a thin-layer chromatography was carried out for each fraction on aluminum plates TLC Silica gel 60 F_254_ (Merck KGaA, Darmstadt, Germany). After fraction 12 was obtained, solvent system I was replaced with solvent system II. Plate chromatography with systems I and II showed that such fractions as 4, 6–8, 10, 12, 14, 16–17, 20–22 and 24 contain basically one component. Fractions 1–3, 5, 9, 11, 13, 15, 18–19, 23 and 25 were mixtures. The retention factor (R_f_) for fractions was represented as 0.45/0.41/0.53/0.67/0.21/0.26/0.29 and 0.35, respectively. The collected fractions were concentrated to colorless, light gray, and bright yellow fine crystalline powders using a rotary evaporator at a temperature of 32–35 °C and a pressure of 0.10–0.13 mbar. The extracts obtained were kept at +5 °C in dark vials under argon medium. The flavonol fractions extracted were identified by ESI and HPLC methods of mass spectrometry. The mass spectrometer with ion trap and atmospheric pressure chemical ionization (APCI) was used, Amazon XBruker Daltonix GmbH (Bremen, Germany) [75].

According to the analysis, the main components of *Aronia melanocarpa* flavonol extract are rutin and quercetin (Table 2 and Table 3). For further studies, rutin and quercetin fractions were dissolved in the estimated amount of 1.3-propylene glycol to prepare a 1% solution. The identification of individual compounds in the extract and fractions was carried out by the HPLC method using such chromatograph as Agilent 1260 Infinity II LC Multiple Wavelength Detector (Agilent Technologies, Inc. Headquarters, Santa Clara, CA, USA) with a multi-wave spectrophotometric detector (Table 2, Figure S1).

HPLC analysis was performed using Zorbax Eclipse C18 (4.6 × 150 mm) and Agilent Hiflex H (250 × 4.6 mm) columns (Agilent Technologies, Inc. Headquarters, Santa Clara, USA). To detect flavonoids, elution was carried out using a gradient method. The mobile phase was a mixture of 0.1% formic acid and acetonitrile solution in the ratios of 1:0, 1:9 and 9:1 as well as a mixture of 0.1% formic acid and acetonitrile solution in the ratios of 1:0, 1:9 and 9:1 for flavonoids. Elute rate was 1.0 mL/min, injection volume was 10 µL, elution time lasted for 30 min, heating unit temperature was 45 °C [76].

Anthocyanins were determined by hydrophilic HPLC method on a reverse phase column; elution was performed by the solution consisting of 30% acetonitrile and 5% formic acid in distilled water. The temperature of the heating unit of the columns was 40 °C; the speed of the mobile phase was 1 mL/min. The input volume was 15 µL.

### 4.7. Chemicals and Reagents

Standards of quercetin, purity ≥ 95.0%; kaempferol, purity ≥ 90.0%; rhamnetin, purity ≥ 95.0%; isorhamnetin, purity ≥ 95.0%; dihydroquercetin (taxifolin), purity ≥ 95.0%; quercetin-3-O-rutinoside (rutin), purity ≥ 94.0%; hesperidin, purity ≥ 80.0%; quercetin-3-O-rhamnoside, purity ≥ 90.0%; quercetin-3-O-galactoside, purity ≥ 90.0%; quercetin-3-O-glucoside, purity ≥ 90.0%; quercetin-3-O-hexoside, purity ≥ 93.0%, were purchased from PhytoLab GmbH & Co. KG, Germany (Merck, Merck KGaA, Darmstadt, Germany) (Figure S4).

Following the method of flavonol production described above, an enriched extract containing flavonols ≥ 88.3% was obtained from *Aronia melanocarpa* berries. In turn, two major compounds, quercetin and rutin with a purity of ≥91.1% and ≥91.2%, respectively, were extracted from it.

### 4.8. Antioxidant Activity and Biologically Active Substances Content in Aronia Extracts

The antioxidant activity of Aronia berries extracts was studied by chemiluminescent and colorimetric methods to determine the ability of substances to interact with free peroxide radicals, namely by 2.2′-azobis (2-amidinopropane) hydrochloride (AAPH) and 2.2-diphenyl-1-picrilhydrazyl (DPPH) respectively. Reagents acquired from Sigma Aldrich, Saint Louis, MO, USA were used for AAPH method; reagents acquired from Alfa Aesar, Saint Louis, MO, USA were used for DPPH method.

The chemiluminescent AAPH assay is described in [77] and adapted to the Lum-1200 luminometer (LLC DISoft, Moscow, Russia) [78]. The results were processed on a personal computer using the PowerGraph 3.3 Professional and OriginLab Pro 9.5 software. In the work of Lissi et al. [79] two approaches to measuring the total antioxidative capacity, taking into account this feature of the curves, are described—the TRAP (total reactive antioxidant potential) method and the TAR (total antioxidant reactivity) method. It is believed that TRAP reflects the amount of antioxidant in the system, and TAR—its activity, i.e., the rate of the antioxidant interaction with radicals. The TRAP method is based on the measurement of the CL latency period. The TAR method was used to determine the value of CL intensity quenching.

The DPPH assay was performed according to the method of Brand-Williams et al. [35] with some modifications. The stock solution was prepared by dissolving 24 mg DPPH with 100 mL ethanol and then stored at −20 °C until needed. The working solution was obtained by mixing 10 mL stock solution with 45 mL ethanol to obtain an absorbance of 1.1 ± 0.02 units at 515 nm using the spectrophotometer. Berries extracts (200 µL) were allowed to react with 800 µL of the DPPH solution for 30 min in the dark. Then the absorbance was taken at 517 nm. Antiradical activity was defined as the amount of antioxidant needed for 50% reduction of the initial concentration of DPPH (effective concentration = EC_50_ ((mol/L) AO/(mol/L) DPPH). The effectiveness of the antioxidant was defined as antiradical power (ARP), defined as 1/EC_50_. The more ARP, the more effective the antioxidant.

### 4.9. In Vitro Tests on the Lymphocyte Cell Line RPMI-1788

The tests were carried out using a conditionally normal human B-lymphocyte cell line, namely RPMI-1788. Lymphoblasts obtained from the Russian collection of cell cultures of D.I. Ivanovskiy Institute of Virology (Moscow). The cells were maintained at 37 °C in RPMI-1640 medium supplemented with 10% fetal bovine serum, 1% essential amino acids and gentamycin antibiotic [80]. Cells were grown at 37 °C with 5% CO_2_.

#### 4.9.1. Determination of Cytoprotective Properties In Vitro

A cell suspension of RPMI-1788 was prepared at a concentration of 10^5^ cells/mL. A 200 µL suspension was pipetted into a 96-well plate and incubated for 24 h. To determine the cytoprotective effect in compliance with [81], immunosuppressive drug CP at a concentration of 1.25 mg/mL was added to the test subjects. Neither extracts nor CP were added to the growth medium of the negative (reference) control during cell cultivation. Only CP was added to the growth medium of positive control cells at an equivalent concentration of 1.25 mg/mL without adding the test substances. Aronia ethanol extract in the concentration range of 2.6–671 μg/mL and a flavonol fraction in concentrations of 6.1–292.5 μg/mL were added to the growth medium of the experimental samples. The cells were again incubated for 24 h. For each group of cells, the cultivation was carried out in triplicate.

#### 4.9.2. Cells Color Staining and Counting

To assess the viability of cell culture, a full growth medium containing also fluorescent dyes was prepared, assuming 198 µL total growth medium, 2 µL Hoechst 33342 (1 mg/mL concentration) and 0.5 µL propidium iodide per well. Further, the cultural medium was replaced with a prepared dye-containing growth medium and incubated for 45 min. After incubation, live and dead cells were counted on a Millipore (Darmstadt, Germany) Guava easyCyte flow cytometer.

### 4.10. In Vivo Experiments

All the procedures were carried out under the ethical guidelines of Kazan (Volga Region) Federal University (protocol No. 4 dated 18 May 2017). The studies were carried out on Wistar laboratory male rats with an induced immune function decrease (immunosuppression) due to cytostatic CP administration. Against the background of induced immunodeficiency in rats, we studied the influence of the Aronia extractives on the immune status of animals, namely, the number of leukocytes and their subpopulation ratio in peripheral blood and the functional state of immune cells (chemiluminescent and phagocytic activity).

Over a period of 7 days, the rats were fed with water (Control Group or Group C), the flavonol fraction obtained from the extract of *Aronia melanocarpa* berries at a dose of 50 mg/kg on a dry basis (Group P), and a solution of quercetin and rutin in concentrations equivalent to the flavonol fraction of the extract (Group Q). The blend of purified flavonol standards—quercetin and rutin was—1.3:1. The dose of the blend was 4.33 mg/kg and was equivalent to the corresponding flavonols in the flavonol fraction of *Aronia melanocarpa* extract (Group P). The volume of introduced solutions and extracts for rats was 0.1 mL per 100 g of mass. Drinking water in the Control Group was fed to create similar exposure conditions as in the experimental groups. 

To induce immunodeficiency, rats were abdominally injected with a single dose of 25 mg/kg on the 8th day of the experiment and continued to feed orally for the next 7 days.

The first blood sampling was performed on the first day, before the test had started. The obtained results were accepted as reference values. After that, we tested the blood again on the 8th day, i.e., one day after CP administration, then on 14 and 21 days, i.e., 7 and 14 days after administration of extracts with induced immunosuppression in the background.

#### 4.10.1. Hematological Studies

To determine the number and subpopulation of the immune cells in peripheral rat blood, a Mythic 18 Vet (Orphee SA, Plan-les-Ouates, Switzerland) automatic hematology analyzer and a special reagent kit were used. The rat blood was tested to determine the total count of white blood cells (WBC), lymphocyte count in absolute units and in relation to the total count of WBC (LYM and LYM%, respectively), monocytes (in a similar way, i.e., MON and MON%) and granulocytes (GRA and GRA%). Red blood cell (RBC) count, hemoglobin (HGB) concentration, platelet (PLT) count and mean platelet volume (MPV) were determined.

#### 4.10.2. Functional State of Neutrophilic Granulocytes in Rats’ Blood

To evaluate the formation of ROS by neutrophils, a recording was made of the own light emission of cells isolated from the peripheral blood of experimental rats using the method described in the work [82], without the use of a CL activator (spontaneous activity), as well as a response signal of neutrophils’ CL to activation by zymosan (induced activity).

On the kinetic curve of the luminol-dependent CL of neutrophils, the following parameters were determined: the time showing peak CL (T_max_, s), maximum luminous intensity (I_max_, c.u.), slew rate of the CL curve (Slope, c.u.) and area under the CL curve (AUC, c.u.) for spontaneous CL (AUC_1_) and CL induced by zymosan (AUC_2_).

The amplification of the induced CL vs. spontaneous CL was determined by the area ratio under the CL curve (AUC_2_/AUC_1_), thus determining the I_act_, i.e., the activation index (c.u.).

The evaluation of phagocytosis, or bactericidal activity, was carried out using cytofluorometry employing the Millipore (Darmstadt, Germany) Guava easyCyte flow cytometer. Inactivated bacteria, *E. coli*, labeled with fluorescein-5-isothiocyanate (FITC) (Sigma Aldrich, Burghausen, Germany), were used as an agent for phagocytosis using the method described in [83,84,85]. The phagocytosis reaction was performed by incubation of the leukoconcentrate (100 µL), obtained by the method described in the work [82] and containing 1 × 10^6^ cells per 1 mL with a suspension of FITC-labelled bacteria *E. coli* (10 µL, the number of bacteria being 5 × 10^7^ per 1 mL), for 30 and 120 min at 37 °C. 

The phagocyte number (PN) of neutrophils for FITC-labeled *E. coli* was calculated as the number of bacterial particles per one phagocytosed neutrophil or monocyte; phagocytic activity (PA)—as the number of active phagocytes absorbed FITC-labeled bacteria, to the total number of monocytes or neutrophils; a phagocyte index (PI)—as the bacteria average per any neutrophil or monocyte; and a phagocytosis completion index (PCI) in c.u. defined as PN ratio in 30 min to PN in 120 min [84,86].

#### 4.10.3. Lipid Peroxidation in Rats’ Blood

The extent of LP was determined by the MDA content in the erythrocytes of rat blood. MDA in blood was determined by carrying out the reaction between the reactive agent and thiobarbituric acid as described by Buege and Aust [87], but with a slight modification of the method. Red blood cells in an amount of 0.1 g washed with normal saline were homogenized into 0.15 mol L^−1^ KCl at a ratio of 1:9 with the help of a glass homogenizer. One volume of RBC was thoroughly mixed with two volumes of a matrix solution of 15% wt./vol. trichloroacetic acid, 0.375% wt./vol. thiobarbituric acid and 0.25 mol × L^−1^ hydrochloric acid. The solution was heated for 15 min over a boiling water bath. After cooling, the sediment was centrifugated at 1000× *g* for 10 min. The absorption of the transparent supernatant was determined at 535 nm and the MDA concentration was calculated using 1.56 × 10^5^ mol^−1^ cm^−1^ as the molar absorption coefficient. The MDA results were expressed in nmol per g of blood with initial moisture.

### 4.11. Statistical Analysis

The data were processed using Microsoft Excel 2016 and OriginPro 9.5 (OriginLab Co., Northampton, MA, USA). Data were compared using a non-parametric Kruskal-Wallis test. The precise *p*-values were calculated for the pair-wise comparisons between the groups using the Mann-Whitney Test. Data analysis employed IBM SPSS 23.0 statistical software (Chicago, IL, USA). The obtained values were represented as mean values ± the standard deviation of the mean. The level of *p* < 0.05 was considered statistically significant.

## 5. Conclusions

In the course of the work, an enriched extract with a flavonol content of ≥88.3% was obtained from the berries of *Aronia melanocarpa* using a modified Darco G-60 sorbent, which made it possible to significantly increase the recovery of flavonol compounds, the degree of purification, and reduce the amount of sorbent used per unit of extracted raw material. The flavonol fraction from the extract of *A*. *melanocarpa* showed a high capability for oxidant neutralization in the examined cell-free systems. The interaction of the extracted flavonols in the fraction revealed a high synergistic effect in vitro in methods of DPPH and luminol-enhanced chemiluminescence. The data from the hematology investigation suggest that Aronia flavonol fraction and individual flavonols exert a minimal impact on the leukogram changes induced by CP.

The capability of the Aronia flavonol fraction to activate monocytes was mainly observed in healthy animals. The administration of quercetin and rutin blends enhances the phagocytosis process, as evidenced by the increase in the phagocytosis completeness index in granulocytes and monocytes, both in healthy and immunosuppressed animals. The mixture of quercetin and rutin was shown to have a positive impact on the “respiratory burst” formation of neutrophilic granulocytes in healthy animals. Meanwhile, in case of induced immunosuppression, the introduction of this blend causes a decrease in the activity of ROS’ products. The introduction of the Aronia flavonol fraction stimulates the formation of the “respiratory burst” of the neutrophil granulocytes both at dormancy and during induction with zymosan. In vivo, the blend of quercetin and rutin as its glycoside leads to a decrease in LP processes, since it reduces the MDA level of the blood of animals to its initial values. Under the same exposure conditions, the flavonol fraction of Aronia exhibits a less pronounced effect on this value.

The studies have revealed good perspectives for the flavonol fraction, mainly quercetin and rutin, extracted from *Aronia melanocarpa*.

## Figures and Tables

**Table 1:** Total content of sugars and natural antioxidants in ethanol and flavonol extracts of *Aronia melanocarpa* berries (based on dry matter).

Materials	pH	Total Sugars ^1^, mg Xy/g Extract	Total Flavonoids ^2^, mg Rut/g Extract	Total Anthocyanins, mg/g Extract
Ethanol extract	7.17–7.29	61.353	39.31	91.715
Flavonol fraction extract	7.15–7.22	0.157	889.63	0.023

^1^ Total sugar content per xylose equivalent (Xy); ^2^ total flavonoids per rutin (Rut) equivalent.

**Table 2:** Individual flavonol quantities identified in the ethanol extract and flavonol fraction of *Aronia melanocarpa* berries.

Sample	Individual Flavonols, mg/g Dry Ext.				
	Quercetin	Kaempferol	Rhamnetin	Isorhamnetin	Dihydroquercetin
Ethanol extract	17.215	0.693	0.561	0.057	2.261
Flavonol fraction extract	365.323	15.635	12.973	1.367	67.363

**Table 3:** Glycosylated flavonol quantities identified in the ethanol extract and flavonol fraction of *Aronia melanocarpa* berries.

Sample	Glycosidic Form of Flavonols, mg/g Dry Ext.					
	Quercetin-3-O-rutinoside	Hesperetin-7-O-rutinoside	Quercetin-3-O-rhamnoside	Quercetin-3-O-galactoside	Quercetin-3-O-glucoside	Quercetin-3-O-hexoside
Ethanol extract	13.591	1.379	0.718	1.423	0.706	0.426
Flavonol fraction extract	315.365	31.236	16.257	32.215	15.976	9.653

**Table 4:** Anthocyanin quantities identified in the ethanol extract and flavonol fraction of *Aronia melanocarpa* berries.

Anthocyanins, mg/g Dry Ext.		Sample	
		Ethanol Extract	Flavonol Fraction Extract
Cyanidin–3–O–galactoside	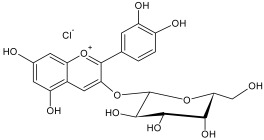	55.093	0.011
Cyanidin–3–O–glucoside	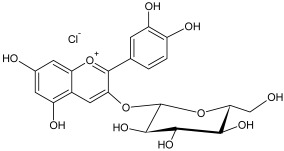	6.57	-
Cyanidin–3–O–arabinoside	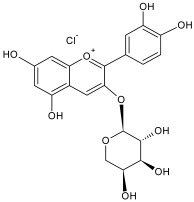	16.44	0.008
Cyanidin–3–O–xyloside	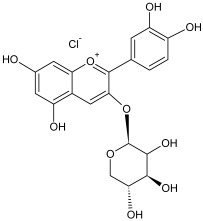	8.91	0.002

**Table 5:** Column chromatography output of individual flavonol fractions.

Fraction	Output Relative to Flavonol Fraction, %	Major Molecular Mass Peak, [M+H]^+^	Mass Fragments, *m*/*z*	The Main Components of the Faction		
				Compound	Structure	Content, mg/g Dry Fraction
4	1.39	287	–	Kaempferol	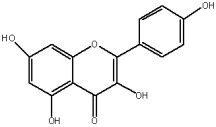	871.32
6–8	31.45	303	–	Quercetin	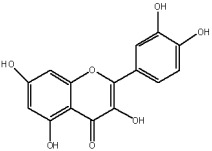	910.9
10	1.23	317	–	Rhamnetin;	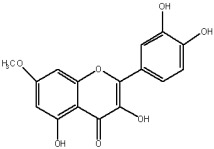	83.74
				Isorhamnetin	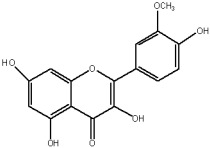	840.49
12	5.98	305	–	Dihydroquercetin	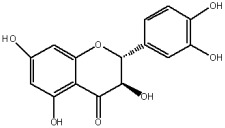	885.6
14	1.41	449	303	Quercetin–3–O-–rhamnoside	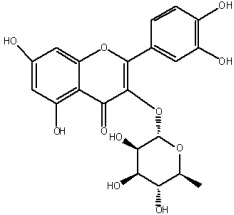	857.91
16–17	5.26	464 and 465	303	Quercetin–3–O–galactoside;	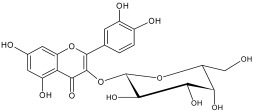	514.52
				Quercetin–3–O–glucoside;	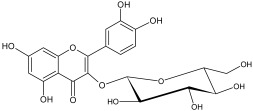	251.09
				Quercetin–3–O–hexoside	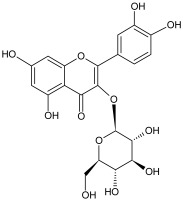	142.08
20–22	28.03	611	303 and 465	Rutin	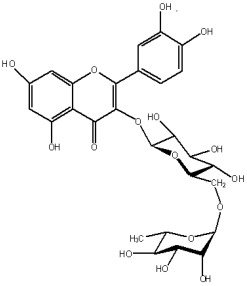	912.36
24	2.71	611	303 and 465	Hesperidin	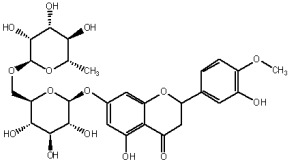	786.12

**Table 6:** Individual flavonol AOA analysis, as well as extracts from *A. melanocarpa* by the DPPH method.

Sample	EC_50_ ^1^, mg/mL	ARP ^2^, mg/mL^−1^
Flavonol fraction of *A. melanocarpa*	0.0026	391.57
Ethanol extract of *A. melanocarpa*	0.0237	42.19
Quercetin	0.0126	79.15
Quercetin-3-O-rutinoside (Rutin)	0.0431	23.18
Quercetin–Rutin blend	0.0265	37.79

^1^ EC_50_—effective concentration for 50% reduction in the initial concentration of DPPH; ^2^ ARP—the effectiveness of the antioxidant defined as 1/EC_50_.

**Table 7:** Comparative analysis of the physical properties of macroporous resins and carbon sorbents.

Sorbent	Adsorption Capacity for Quercetin, mg/g	Desorption Coefficient, %	Selectivity for Flavonols, %
Darco G-60	115 ± 1.13	57.31 ± 2.35	71.37
Darco G-60 after activation	323 ± 3.27	85.37 ± 3.51	89.35
X-5	53.8 ± 1.56	82.99 ± 2.01	79.93
D4020	45.77 ± 0.50	72.44 ± 0.16	67.31
D101	45.33 ± 2.06	72.68 ± 3.13	65.25
LS-305	45.78 ± 0.78	54.22 ± 2.33	75.33
LS-46D	42.82 ± 0.08	56.11 ± 2.02	76.21
NKA-9	51.26 ± 1.68	78.33 ± 1.69	53.87

**Table 8:** Sorption parameters of carbon before and after activation.

Carbon Sample	Iodine Number, mg/g	Concentration of COOH Groups, mmol·g^−1^	Sorption Capacity, mg/g	
			By Quercetin	By Rutin
Darco G-60	700	0.117	115	131
Darco G-60 after activation	890	0.289	323	355

## Data Availability

The data presented in this study are available upon request from the corresponding author.

## Supplementary Materials

The following supporting information are available online at: https://www.mdpi.com/article/10.3390/plants12162976/s1, Figure S1: HPLC chromatograms of fractions. (A) Fraction 4; (B) fractions 6–8; (C) fraction 10; (D) fraction 12; (E) fraction 14; (F) fractions 16–17; (G) fractions 20–22; (H) fraction 24; Figure S2: electrospray ionization (ESI) mass spectra of flavonol fractions. (A) Fraction 4; (B) fractions 6–8; (C) fraction 10; (D) fraction 12; (E) fraction 14; (F) fractions 16–17; (G) fractions 20–22; (H) fraction 24; Figure S3: values of spontaneous and zymosan-activated chemiluminescence of rat neutrophils: (A) chemiluminescence intensity (I_max_, c.u.), (B) the time showing peak CL (T_max_, s), (C) slew rate of the chemiluminescence curve (Slope). * *p* < 0.05, significance of differences compared to the first day of the experiment, ° *p* < 0.05, significance of differences between the animal groups; Figure S4: HPLC chromatograms of standard compounds. (A) Individual flavonols; (B) glycosidic form of flavonols.
