# Supplementary material for: Aronia melanocarpa Flavonol Extract—Antiradical and Immunomodulating Activities Analysis
Source: Plants (Basel). 2023 Aug 17;12(16):2976. doi: 10.3390/plants12162976 (PMC10458422; doi:10.3390/plants12162976)
Supplement: Supplementary file 1 [file plants-12-02976-s001.zip › plants-2541245-supplementary.pdf]

Figure S1: HPLC chromatograms of fractions. (A) Fraction 4; (B) fractions 6-8; (C) fraction 10; (D) fraction 12; (E) fraction 14; (F) fractions 16-17; (G) fractions 20-22; (H) fraction 24.

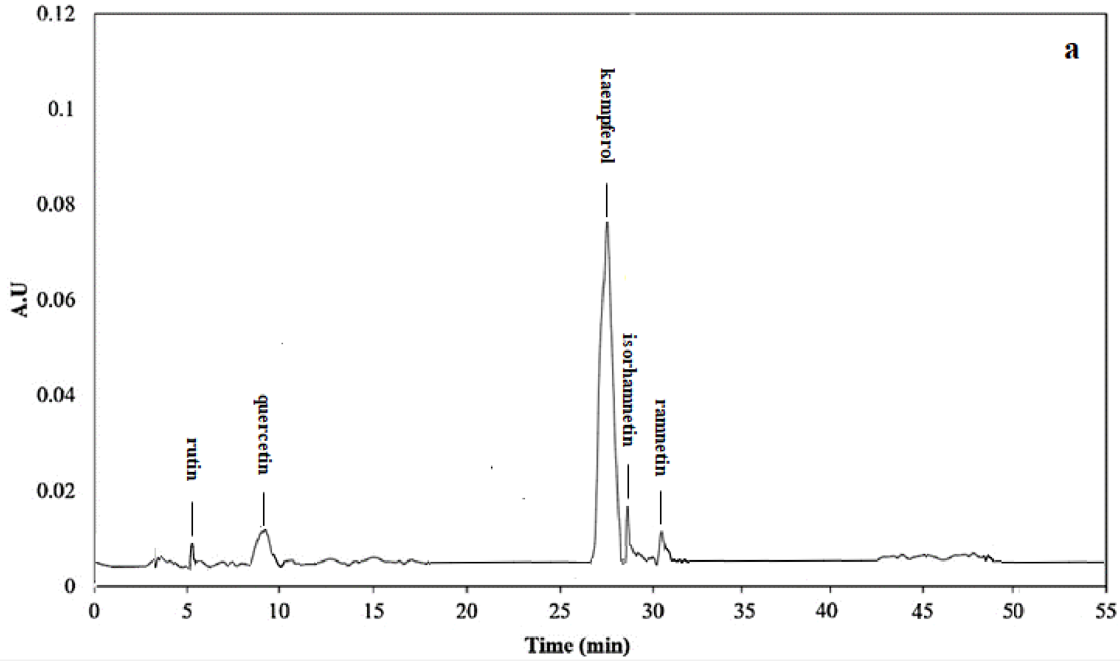

(A)

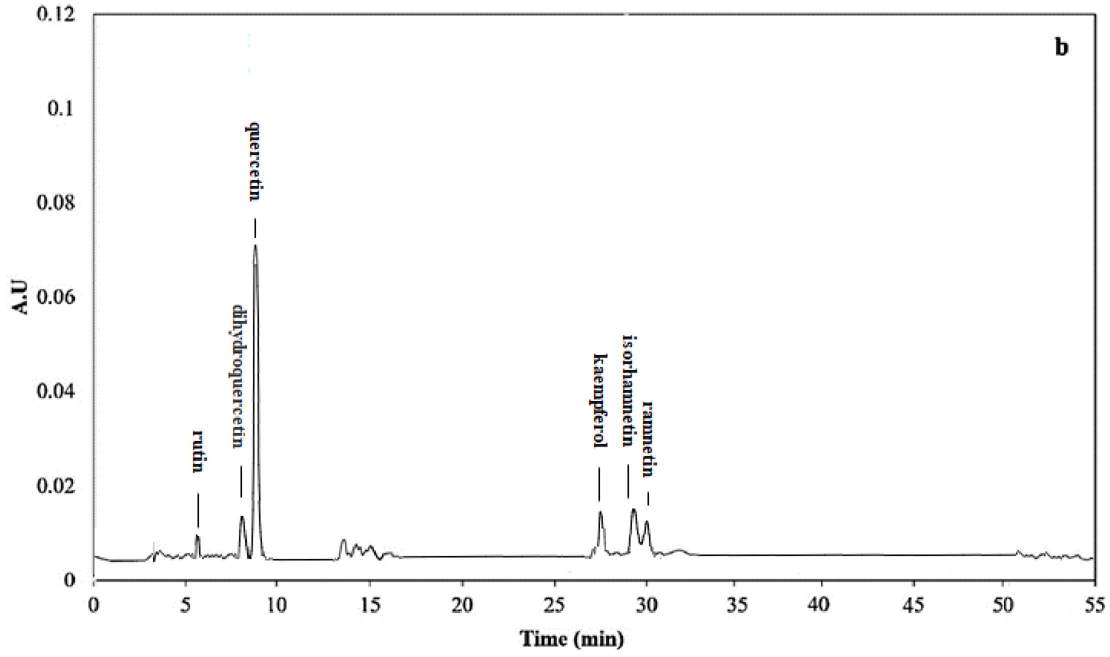

(B)

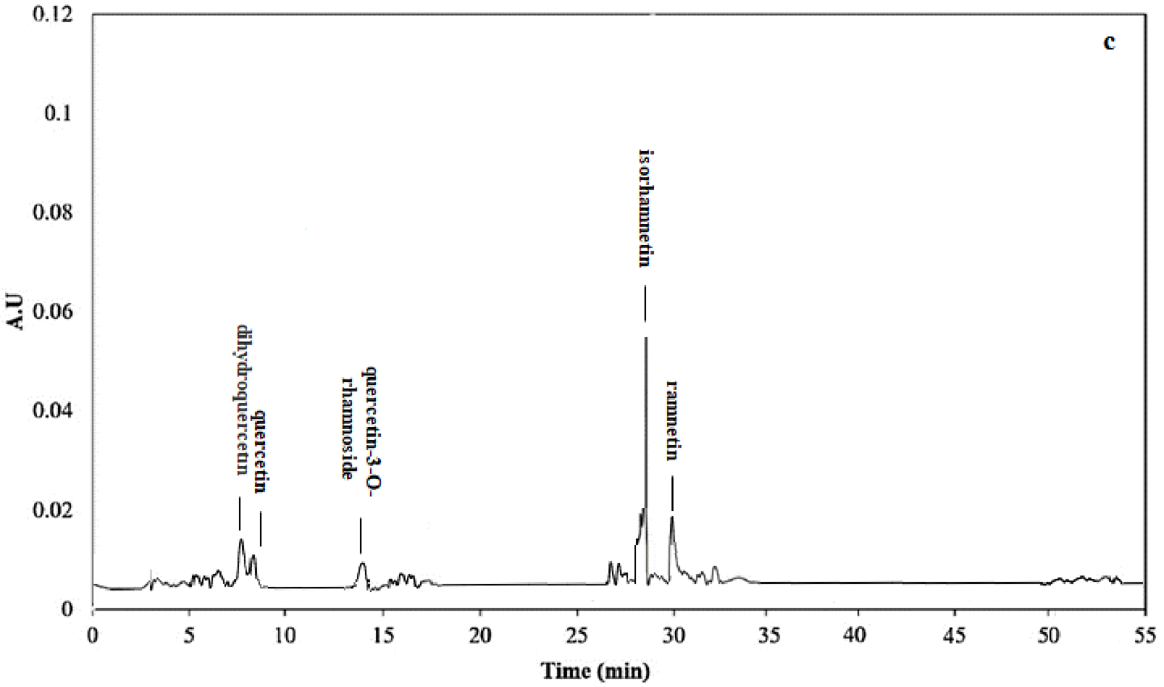
c
A.U
Time (min)
dihydroquercetin
quercetin
quercetin-3-O-rhamnoside
isorhamnetin
ramnetin
(C)

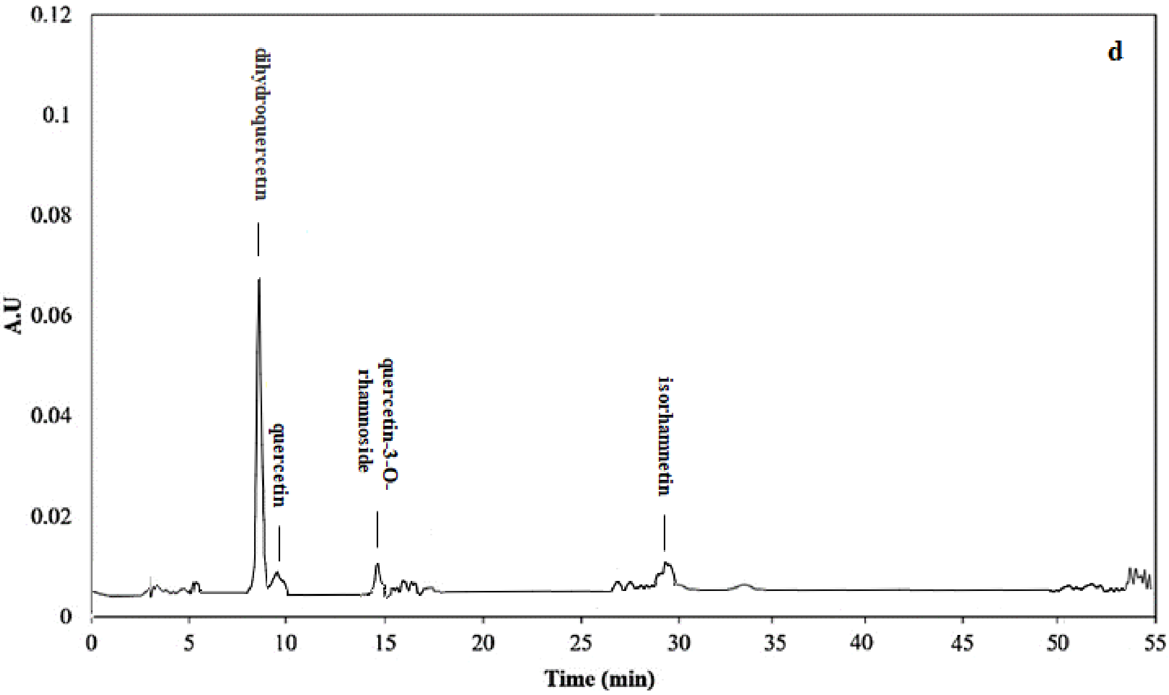
d
A.U
Time (min)
dihydroquercetin
quercetin
quercetin-3-O-rhamnoside
isorhamnetin
(D)

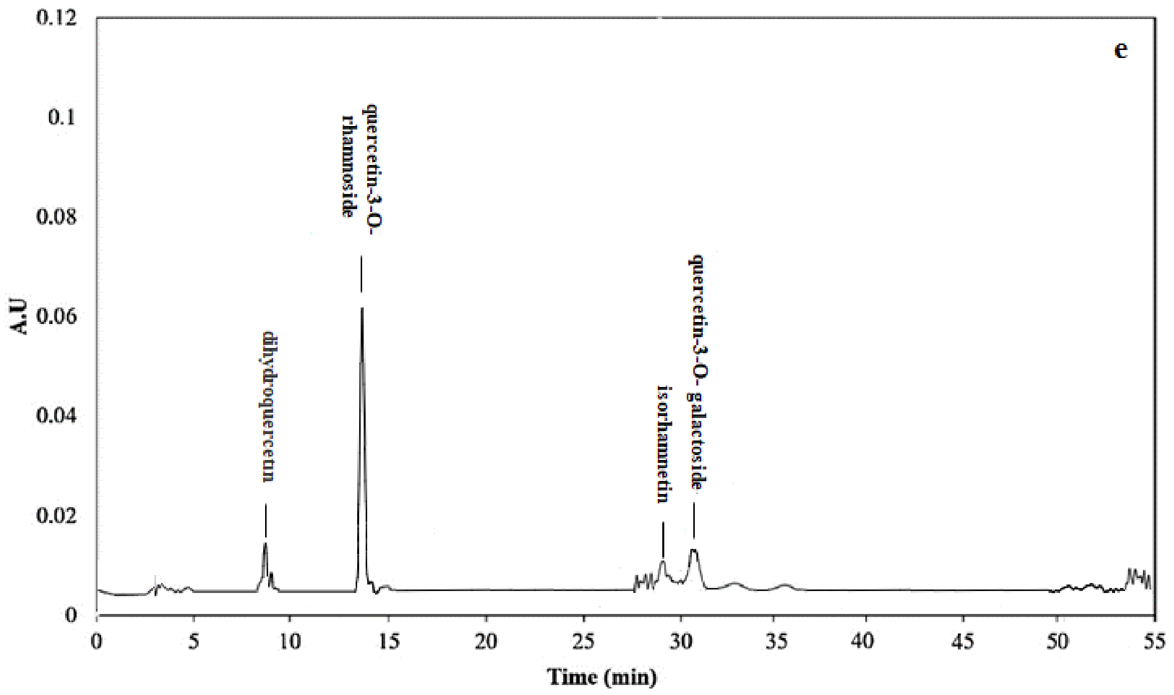

e
A.U
0.12
0.1
0.08
0.06
0.04
0.02
0
dihydroquercetin
quercetin-3-O-rhamnoside
isorhamnetin
quercetin-3-O- galactoside
0
5
10
15
20
25
30
35
40
45
50
55
Time (min)

(E)

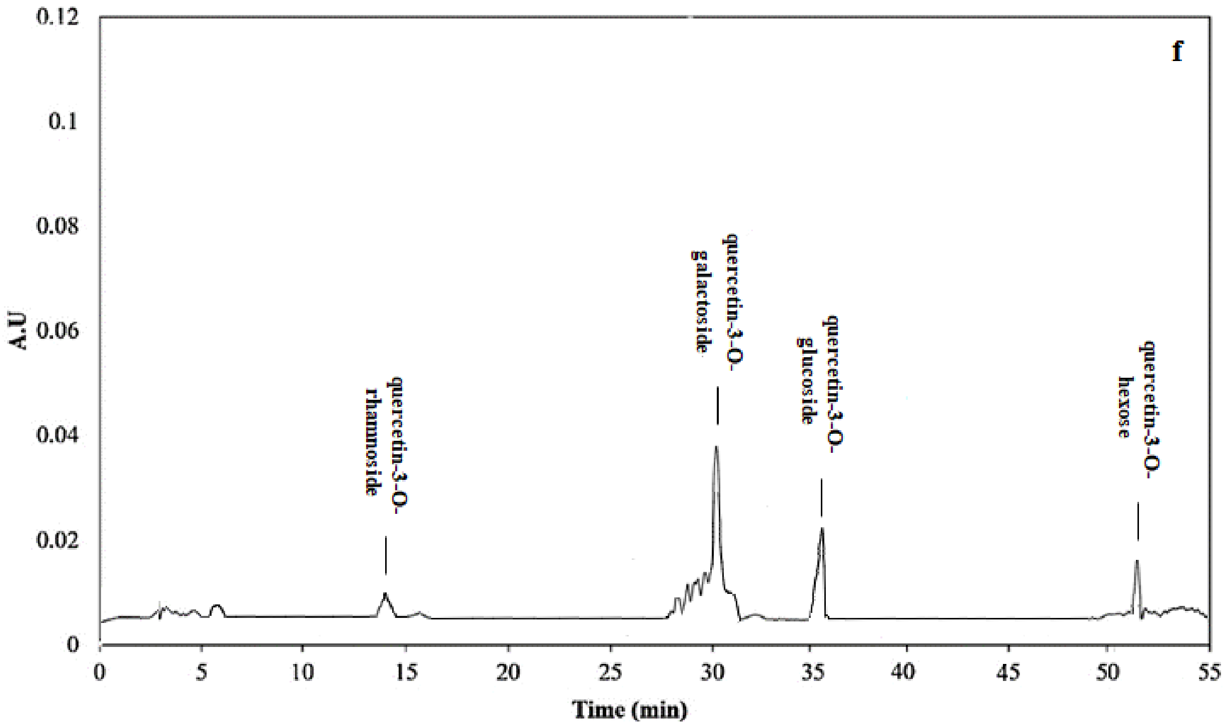

f
A.U
0.12
0.1
0.08
0.06
0.04
0.02
0
quercetin-3-O-rhamnoside
quercetin-3-O-galactoside
quercetin-3-O-glucoside
quercetin-3-O-hexose
0
5
10
15
20
25
30
35
40
45
50
55
Time (min)

(F)

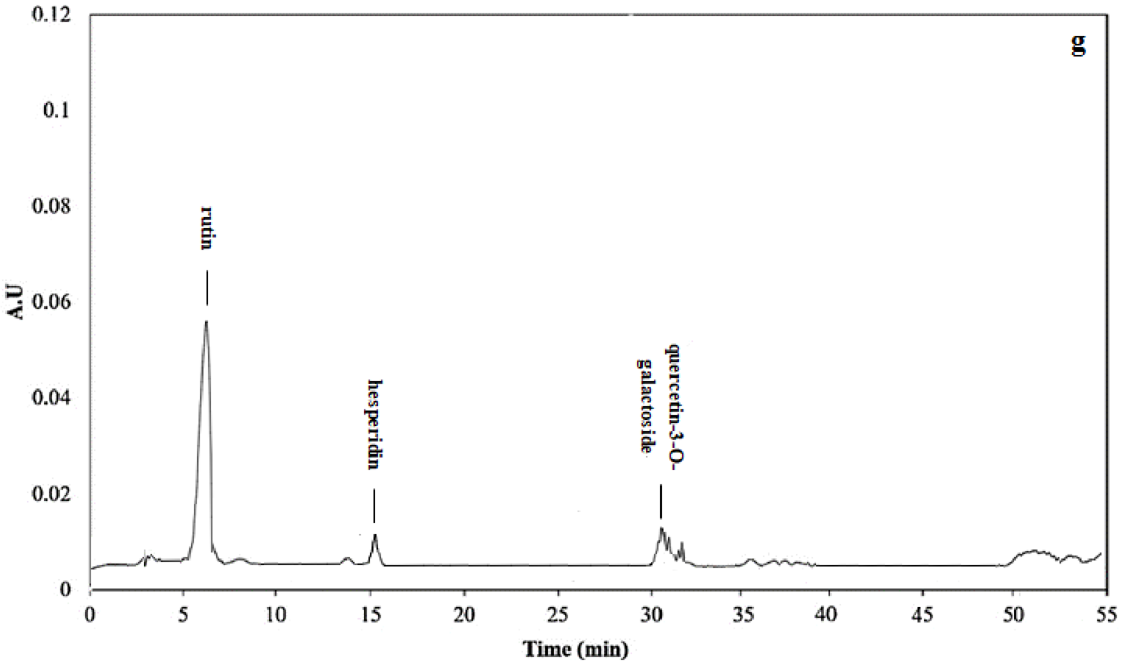

g
A.U
0.12
0.1
0.08
0.06
0.04
0.02
0
rutin
hesperidin
quercetin-3-O-galactoside
0
5
10
15
20
25
30
35
40
45
50
55
Time (min)

(G)

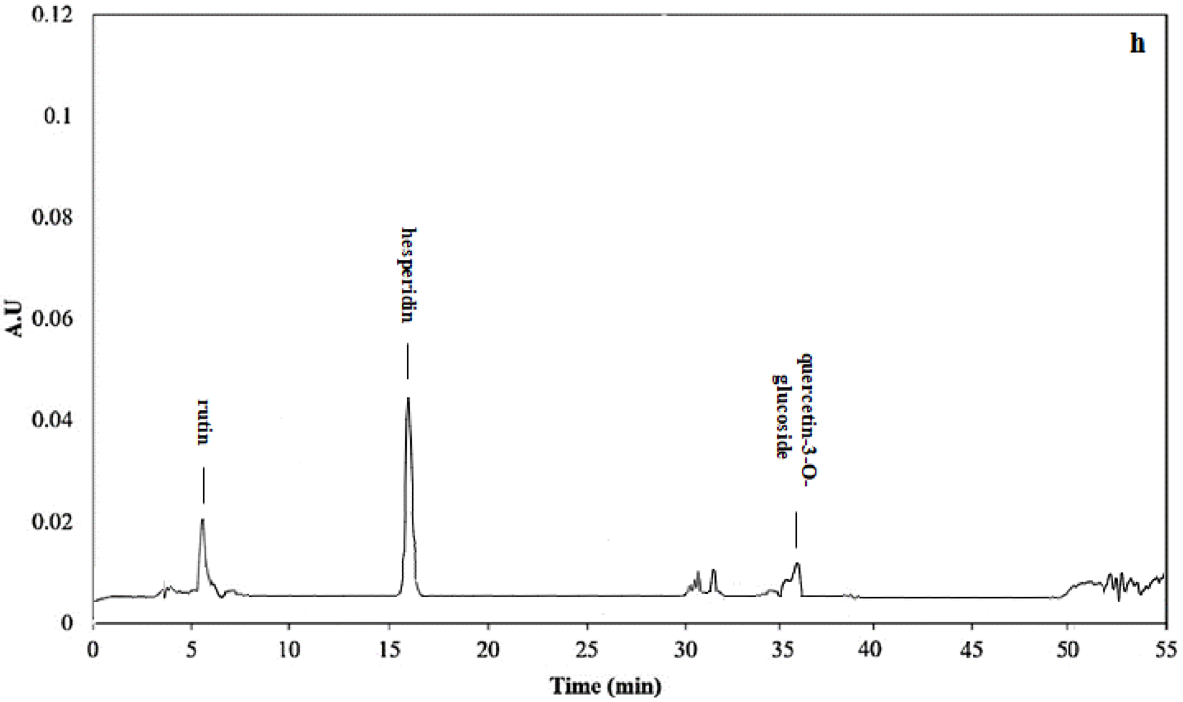

h
A.U
0.12
0.1
0.08
0.06
0.04
0.02
0
rutin
hesperidin
quercetin-3-O-glucoside
0
5
10
15
20
25
30
35
40
45
50
55
Time (min)

(H)

Figure S2: Electrospray ionization (ESI) mass spectra of flavonol fractions. (A) Fraction 4; (B) fractions 6-8; (C) fraction 10; (D) fraction 12; (E) fraction 14; (F) fractions 16-17; (G) fractions 20-22; (H) fraction 24.

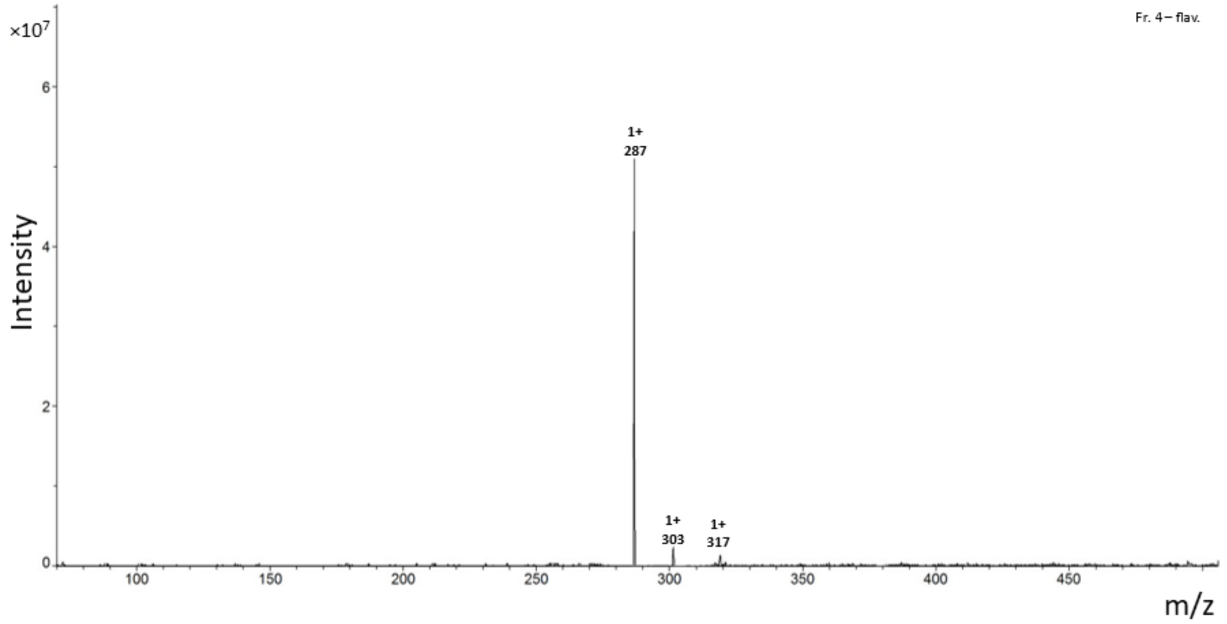

(A)

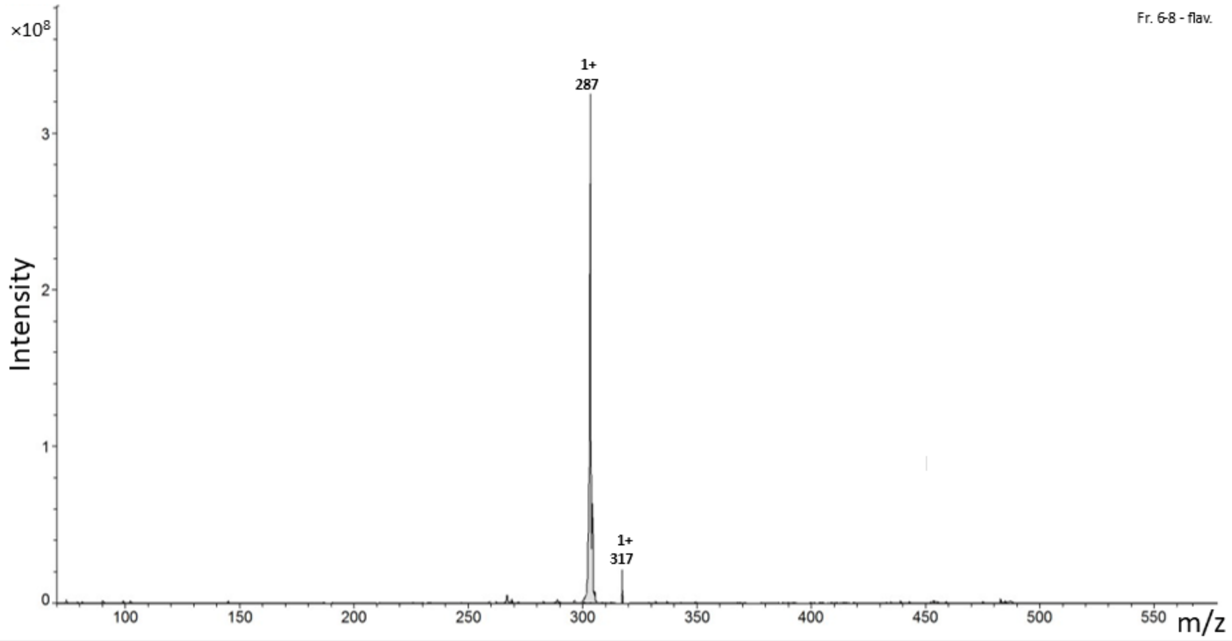

(B)

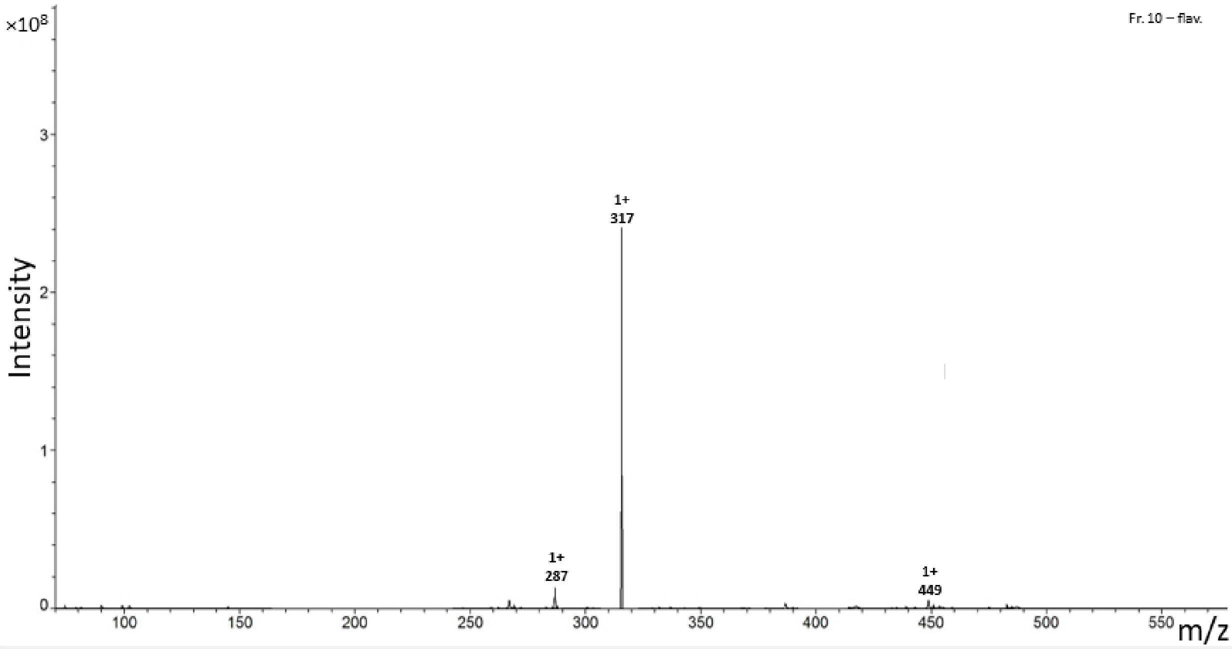
×10^8
Fr. 10 – flav.
Intensity
3
2
1
0
1+
317
1+
287
1+
449
100
150
200
250
300
350
400
450
500
550
m/z

(C)

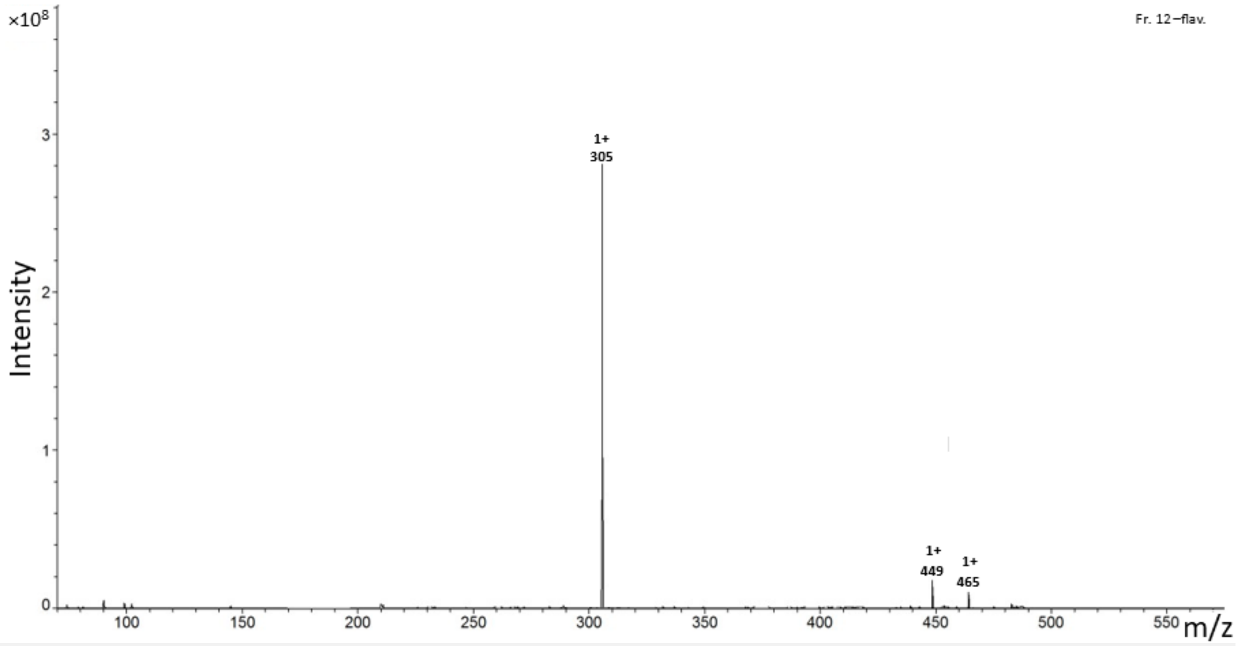
×10^8
Fr. 12 –flav.
Intensity
3
2
1
0
1+
305
1+
449
1+
465
100
150
200
250
300
350
400
450
500
550
m/z

(D)

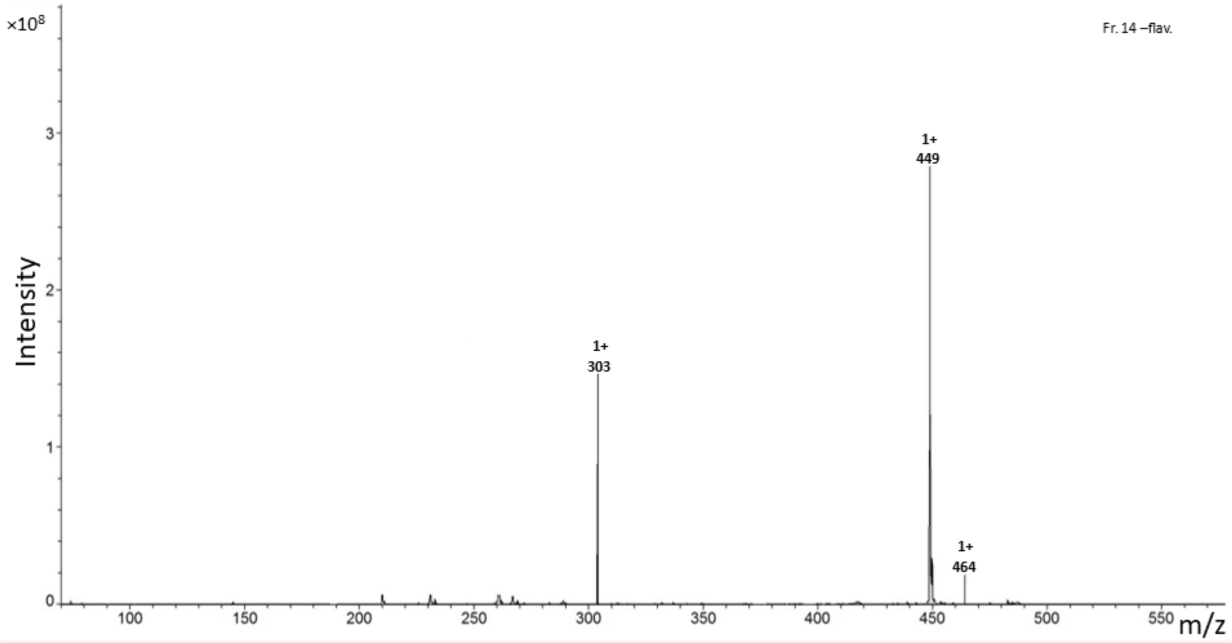
×10^8
Fr. 14 –flav.
Intensity
1+
449
1+
303
1+
464
0
1
2
3
100
150
200
250
300
350
400
450
500
550
m/z

(E)

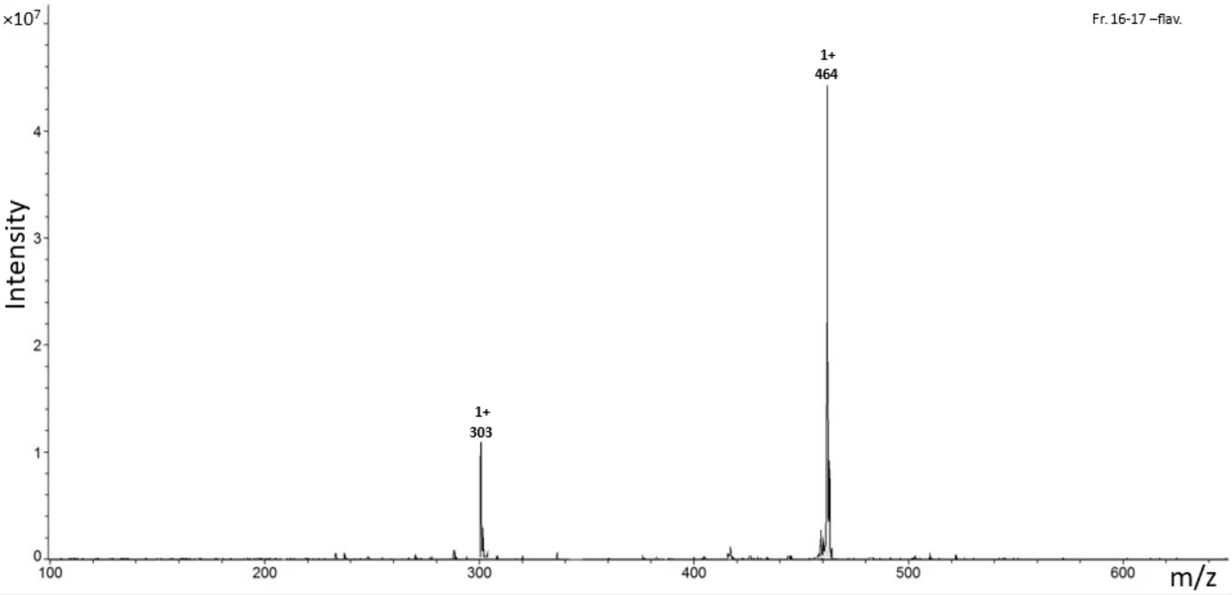
×10^7
Fr. 16-17 –flav.
Intensity
1+
464
1+
303
0
1
2
3
4
100
200
300
400
500
600
m/z

(F)

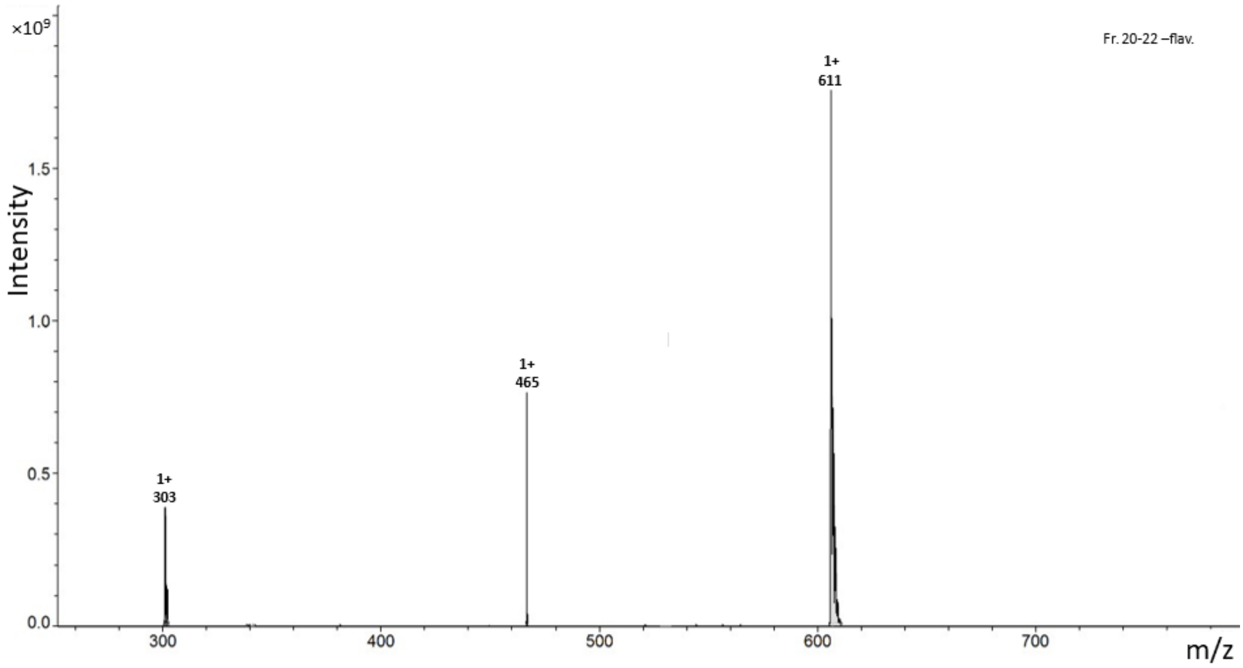
×10^9
Fr. 20-22 –flav.
Intensity
1.5
1.0
0.5
0.0
1+
611
1+
465
1+
303
300
400
500
600
700
m/z

(G)

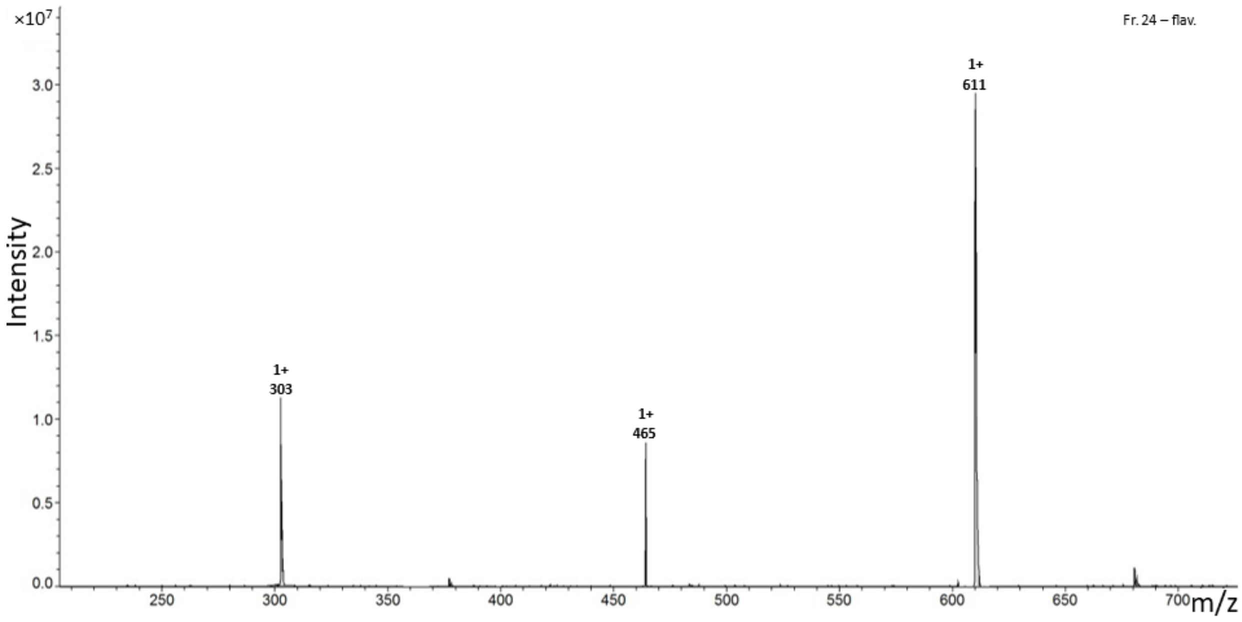
×10^7
Fr. 24 – flav.
Intensity
3.0
2.5
2.0
1.5
1.0
0.5
0.0
1+
611
1+
303
1+
465
250
300
350
400
450
500
550
600
650
700
m/z

(H)

Figure S3: Values of spontaneous and zymosan-activated chemiluminescence of rat neutrophils: (A) chemiluminescence intensity (I_{max}, c.u.), (B) the time showing peak CL (T_{max}, s), C – slew rate of the chemiluminescence curve (Slope, c.u.). * $p < 0.05$, significance of differences compared to the first day of the experiment, ° $p < 0.05$, significance of differences between the animal groups.

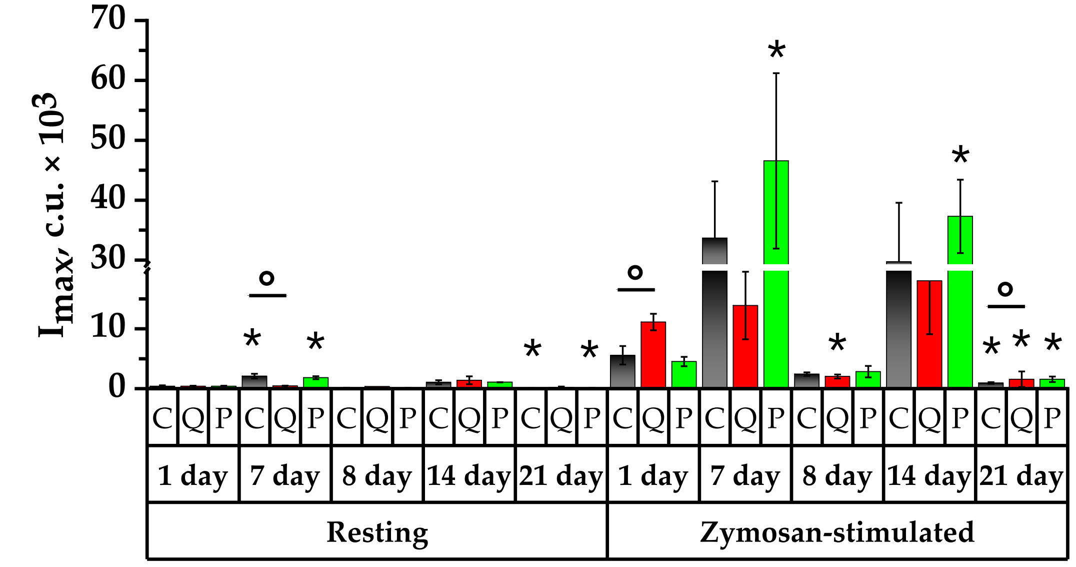

(A)

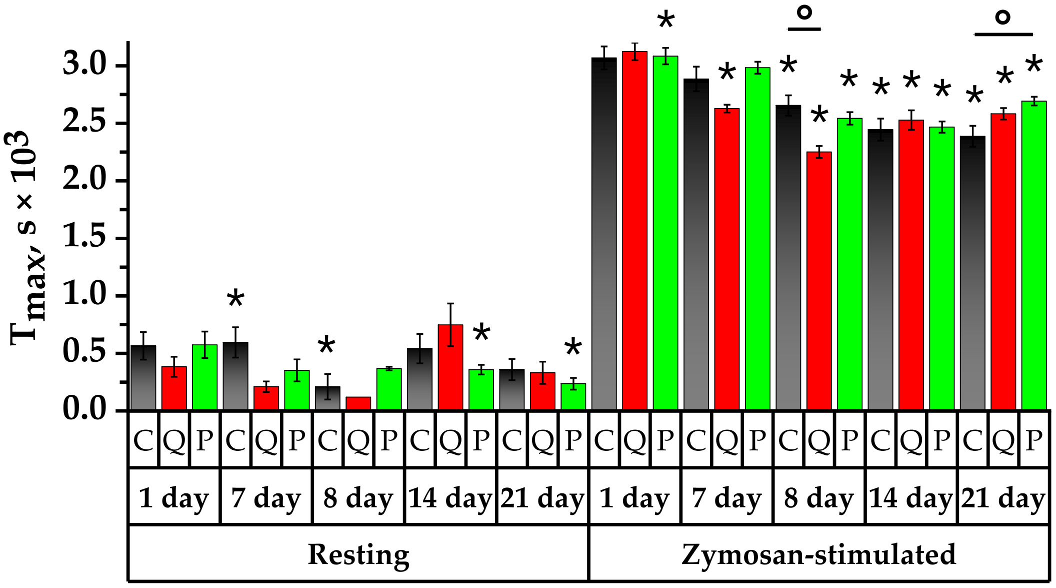

(B)

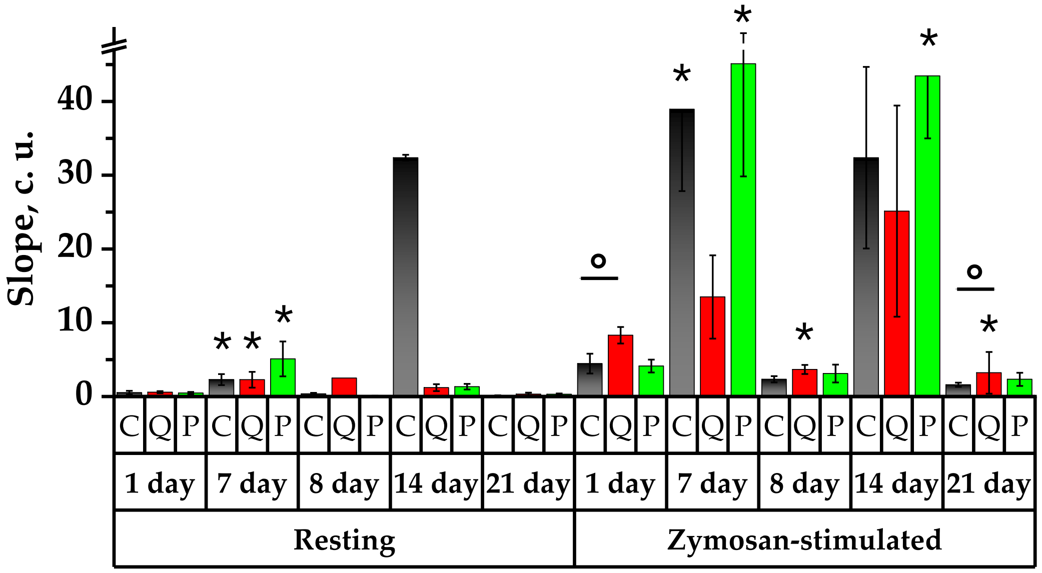

Slope, c. u.
0
10
20
30
40
C Q P C Q P C Q P C Q P C Q P C Q P C Q P C Q P C Q P C Q P
1 day
7 day
8 day
14 day
21 day
1 day
7 day
8 day
14 day
21 day
Resting
Zymosan-stimulated

(C)

Figure S4: HPLC chromatograms of standard compounds. (A) Individual flavonols; (B) glycosidic form of flavonols.

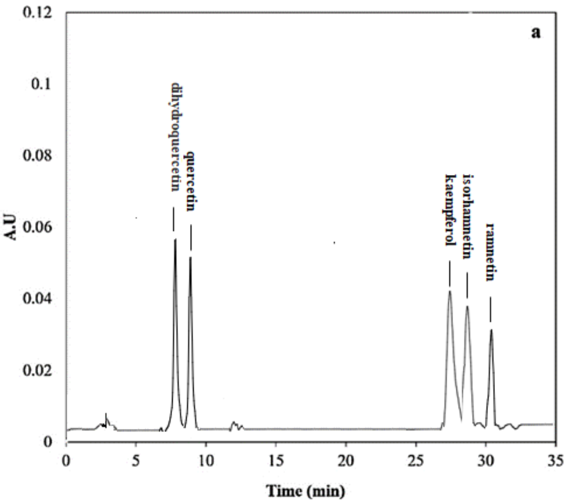

(A)

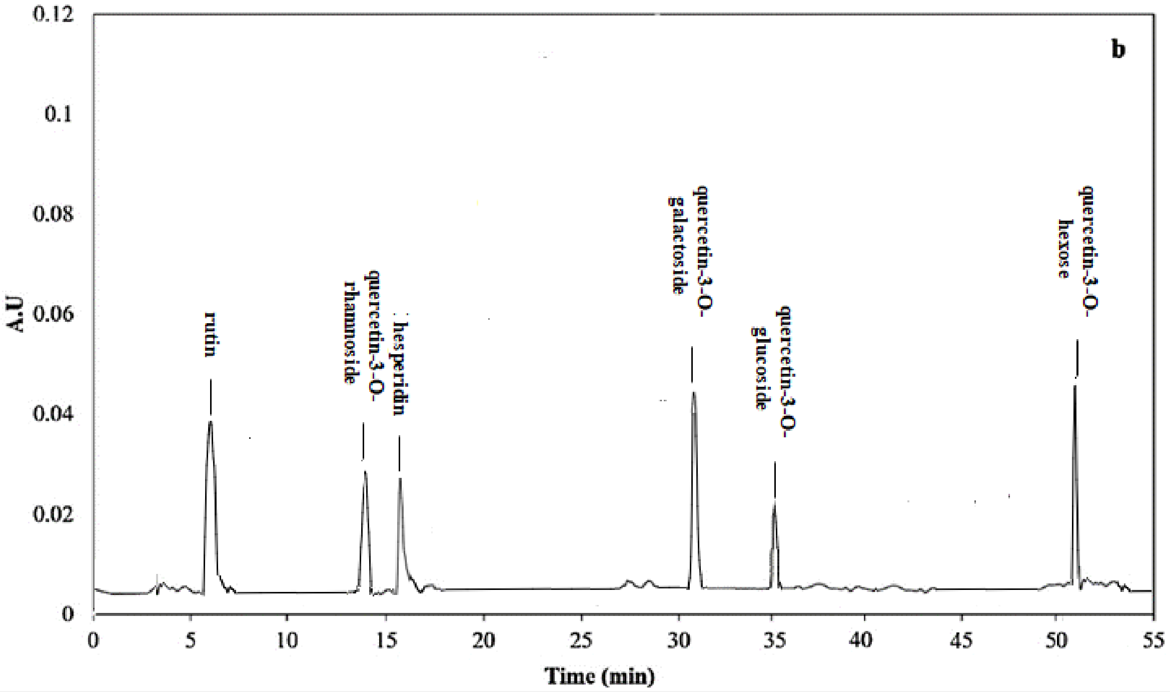

(B)
